# Alkylthienyl Side Groups in Conjugated Polymers Enable Localization of π Electron Density and Facilitate Efficient Hole Transfer

**DOI:** 10.1002/advs.202516088

**Published:** 2026-01-26

**Authors:** Hristo Ivov Gonev, Daniel G. Congrave, Junjun Guo, Jose Marin‐Beloqui, Max Allen, David Bacon, Ankita Kumari, Mohammad Saeed Shadabroo, He Zhu, Dibyajyoti Ghosh, Rachel C. Kilbride, Safa Shoaee, Hugo Bronstein, Tracey M. Clarke

**Affiliations:** ^1^ Department of Chemistry University College London London United Kingdom; ^2^ Department of Chemistry University of Cambridge Cambridge United Kingdom; ^3^ Industrial Catalysis Centre, Department of Chemical Engineering Tsinghua University Beijing China; ^4^ Department of Physical Chemistry Faculty of Science University of Malaga Málaga Spain; ^5^ School of Mathematics and Physical Sciences University of Sheffield Sheffield United Kingdom; ^6^ Department of Materials Science and Engineering and Department of Chemistry Indian Institute of Technology New Delhi India; ^7^ Optoelectronics of Disordered Semiconductors, Institute of Physics and Astronomy University of Potsdam Potsdam‐Golm Germany; ^8^ Department of Physics University of Warwick Coventry United Kingdom; ^9^ XMaS, The UK X‐Ray Materials Science Facility European Synchrotron Radiation Facility Grenoble France; ^10^ Heterostructure Semiconductor Physics Paul‐Drude‐Institut für Festkörperelektronik (PDI) Hausvogteiplatz 57 Berlin Germany

**Keywords:** non‐fullerene acceptors, organic photovoltaics, spectroscopy, triplet state

## Abstract

Recent advances in molecular design have seen peripheral alkoxy groups replaced with alkylthienyl substituents in conjugated polymers for high‐performing organic photovoltaics. However, little is known about the mechanistic origins behind this improvement in performance. In this work, transient absorption spectroscopy is used in conjunction with resonance Raman spectroscopy to shed light on this question. Alkoxy‐substituted polymer PBDB is compared with alkylthienyl‐substituted PBDB‐T in blends with two different acceptors. Larger charge photogeneration yields are observed for PBDB‐T:ITIC‐Th compared to the PBDB:ITIC‐Th blend due to more efficient hole transfer. However, an active triplet formation mechanism via charge recombination leaves the two blends with similar polaron populations on longer timescales. Importantly, resonance Raman spectroscopy demonstrates that the alkylthienyl groups in PBDB‐T enable a stronger coupling of the S_1_ state to the benzodithiophene unit compared to the benzodithiophene‐dione unit. The observation that this localization of electron density on the benzodithiophene unit only occurs for PBDB‐T, and most prominently in the presence of the ITIC‐Th, suggests a strong interaction between the PBDB‐T and ITIC‐Th. This interaction may facilitate the more efficient hole transfer and greater charge photogeneration yields observed for PBDB‐T:ITIC‐Th. These results demonstrate the mechanistic origin of a valuable structure‐function relationship.

## Graphical abstract

Resonance Raman spectroscopy is used in combination with other spectroscopic and morphology characterization techniques to unveil the mechanistic origin of the shift in synthetic strategy to alkylthienyl peripheral groups for high‐performing organic photovoltaics.

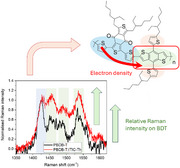

## 1 Introduction

Organic photovoltaic (OPV) devices are currently experiencing a renaissance, recently reaching a landmark efficiency of 20% [1, 2, 3]. Since the early days of simple homopolymers and fullerenes, the community has built upon decades of molecular design knowledge, crafting the complex library of highly efficient materials known today. One notable example is the development of non‐fullerene acceptors (NFAs). While the strong electron affinity of fullerenes kept them at the forefront of OPV development for several years, NFAs offer numerous advantages, including high absorptivity, synthetic tunability and improved electron mobility [4, 5]. Furthermore, there have been crucial advances in the design of conjugated polymers used in OPVs. One of the early advances was the incorporation of alternating electron‐rich (donor, D) and electron‐poor (acceptor, A) groups within the conjugated backbone: essentially replacing homopolymers with co‐polymers. Energy level engineering via judicious selection of electron‐donating and electron‐withdrawing peripheral functional groups has also provided a significant tool in the molecular design arsenal. One of the recent key structural changes has been the replacement of peripheral alkoxy functional groups with alkylthienyl substituents, such as those present in high‐performing archetypical polymers D18 and PM6. The observation of this shift in synthetic strategy and subsequent increase in performance, particularly when blended with NFAs, is largely anecdotal. Nevertheless, we know little about the impact of the polymer's alkylthienyl group in NFA blends, particularly from a mechanistic point of view [6, 7]. Our motivation is therefore to explore the origin of why alkylthienyl substituents produce better performing polymers compared to alkoxy substituents, with a particular focus on NFA blends.

**FIGURE 1:**
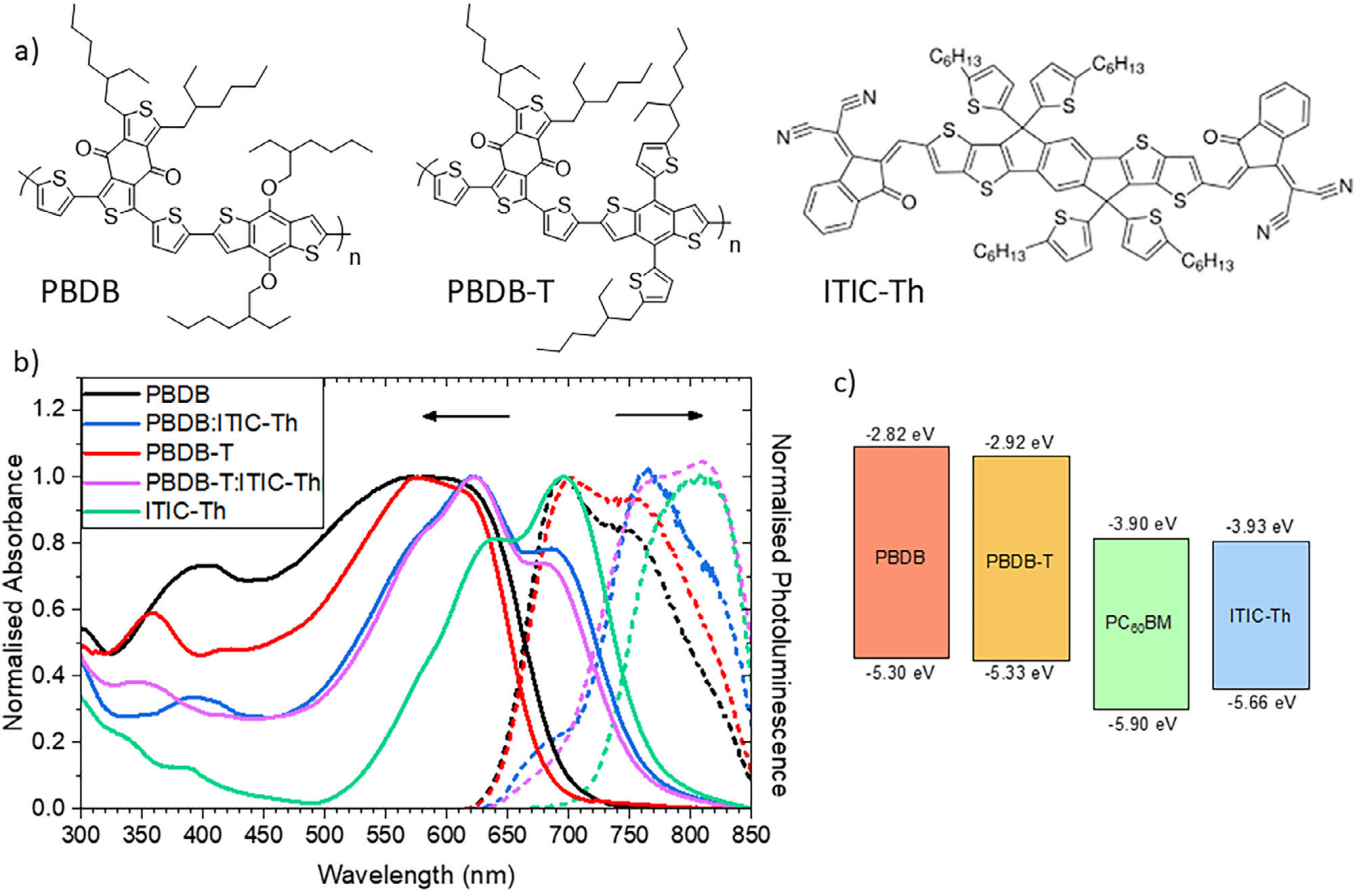
(a) Structures of the materials used in this work. (b) Comparison between the normalized absorbance (solid lines) and emission data (dashed lines) for the pristine films as well as the ITIC‐Th blend films (1:0.8 weight ratio). For the emission spectra, an excitation wavelength of 600 nm was used. (c) HOMO and LUMO energy levels of polymer donors PBDB and PBDB‐T, as well as acceptor materials PC60BM and ITIC‐Th, measured by cyclic voltammetry.

In this paper, a range of spectroscopy techniques is used to explore the photophysics and charge generation dynamics in two polymer analogues: one with alkylthienyl side groups and the other with alkoxy groups, both blended with a representative fullerene and NFA. The well‐known polymer PBDB‐T has been chosen for this study in addition to its less‐studied alkoxy analogue, PBDB. Each of the two polymers is investigated in blends with fullerene acceptor PC60BM and NFA ITIC‐Th. Structures are shown in Figure 1a. ITIC‐Th is the NFA of choice for this study due to its stability [8], greater optical absorption and enhanced electron mobility compared to commonly used ITIC [9, 10]. ITIC‐Th also shows reduced d‐spacing and increased coherence length of π–π stacking as well as a much higher degree of crystallinity relative to ITIC [11]. Transient absorption spectroscopy across multiple timescales is used to delve into excited state mechanisms and photophysics for the four blends, while resonance Raman spectroscopy offers unique insights into the links between molecular and electronic structure [12].

The peripheral alkylthienyl groups in PBDB‐T enable extended π‐conjugation from the polymer backbone into the peripheral thiophene rings. In the PBDB‐T:ITIC‐Th blend, resonance Raman spectroscopy demonstrates that this additional π‐conjugation alters the polymer S_1_ state to become more centered on the benzodithiophene (BDT) donor unit (rather than the benzodithiophene‐dione (BDD) acceptor unit). PBDB, which lacks this additional π‐conjugation, does not show the same effect. The observation that this phenomenon occurs most strongly in the presence of the ITIC‐Th may suggest a close interaction between polymer and NFA. This is consistent with photoluminescence (PL) and atomic force microscopy (AFM) data, which both indicate greater miscibility between PBDB‐T and ITIC‐Th (compared to PBDB and ITIC‐Th). Furthermore, the localization of the polymer S_1_ state on the BDT unit for PBDB‐T:ITIC‐Th may also suggest that the presence of the ITIC‐Th influences the packing in the bulk polymer domains, and this is supported by GIWAXS data. The redistribution of electron density onto the BDT unit in PBDB‐T:ITIC‐Th may facilitate the more efficient hole transfer and thus greater charge generation yield that is observed compared to PBDB:ITIC‐Th. PBDB‐T:ITIC‐Th does not show higher charge populations on longer timescales, however, and this was correlated with increased recombination to form more triplet states. This study, therefore, provides more crucial evidence that efficient charge photogeneration goes hand in hand with efficient triplet formation in polymer/NFA blends. Furthermore, in identifying correlations between the presence of the alkylthienyl group (and consequent localization of electron density on the BDT unit) and enhanced hole transfer, it also provides a vital structure‐function relationship for future molecular design.

## 2 Results and Discussion

### 2.1 UV/Vis and Photoluminescence (PL)

The normalized ground state absorbance spectra of the pristine and blend films are shown in Figure 1b and Figure S1. As expected from their similar bandgaps (Figure 1c) [7], the neat PBDB and PBDB‐T films both have their 0‐0 absorption at ∼625 nm, previously assigned to an intramolecular charge transfer excitation [13]. Addition of the ITIC‐Th or PC60BM acceptor induces morphology changes in the films, which are reflected by shifts in the absorption maxima and alterations to vibronic ratios. For example, it can be observed that the PBDB‐T:PC60BM blend exhibits an increase in the relative amplitude of the 0–0 vibronic band (compared to the 0–1) and an 8 nm red‐shift of the absorption onset relative to pristine PBDB‐T. This is an indication of fullerene‐induced ordering in PBDB‐T:PC60BM, a phenomenon previously seen in other polymer blends, such as PffBT4T‐C9C13 [14] and PBTTT [15]. In contrast, the PBDB:PC60BM film shows no change in vibronic ratio and a small blue‐shift of the absorption onset, suggesting that no increase in order is present. Interestingly, the two ITIC‐Th blend absorption spectra are very similar in the 520–600 nm range, where the polymer absorbs. The overlap between polymer and NFA absorbances makes judgments on relative order difficult.

Figure 1b and Figure S1 show the normalized steady‐state PL data, measured with an excitation wavelength of 600 nm. This wavelength aligns with the absorption maximum of the two polymers, but some NFA absorption is also present. In the pristine polymer films, two emission peaks can be observed at 700 and 760 nm, while pristine ITIC‐Th emits at ∼810 nm (noting the detector limit nearby at 850 nm). The two ITIC‐Th blend films clearly show contributions from both polymer and NFA, but the relative proportions are quite different. PBDB‐T:ITIC‐Th shows a significantly larger contribution from the ITIC‐Th PL at 800–850 nm relative to the polymer PL, as compared to PBDB:ITIC‐Th. Since the polymer absorption at the excitation wavelength is the same in both cases, this may indicate more efficient singlet energy transfer from the polymer to the NFA. There is a good overlap between the emission spectrum of the polymer donor and the absorption spectrum of the ITIC‐Th (especially in the 500–700 nm wavelength range), which is advantageous for Förster resonance energy transfer (FRET) of singlet excitons from polymer to NFA. FRET is common in NFA blends upon polymer excitation, where it causes hole transfer to be the dominant process in exciton separation and increases the power conversion efficiencies [16, 17, 18, 19, 20, 21, 22, 23, 24]. Furthermore, when considering the PL spectra corrected for absorbance (Figure S2), it can be observed that the PL amplitude of the PBDB‐T:ITIC‐Th is less than that of PBDB:ITIC‐Th for both polymer and acceptor PL contributions. As such, it is likely that overall exciton quenching in PBDB‐T:ITIC‐Th is more efficient. This could have its origins in a greater miscibility between PBDB‐T and ITIC‐Th, leading to smaller domain sizes, or longer exciton lifetimes in the case of PBDB‐T:ITIC‐Th enabling more excitons to reach an interface and be quenched. A similar observation is made for the fullerene films: the PL is dominated by the polymer, as expected, and the PBDB‐T:PCBM blend exhibits stronger quenching (98%) than the PBDB:PCBM blend (82%).

### 2.2 Morphology Characterization

**FIGURE 2:**
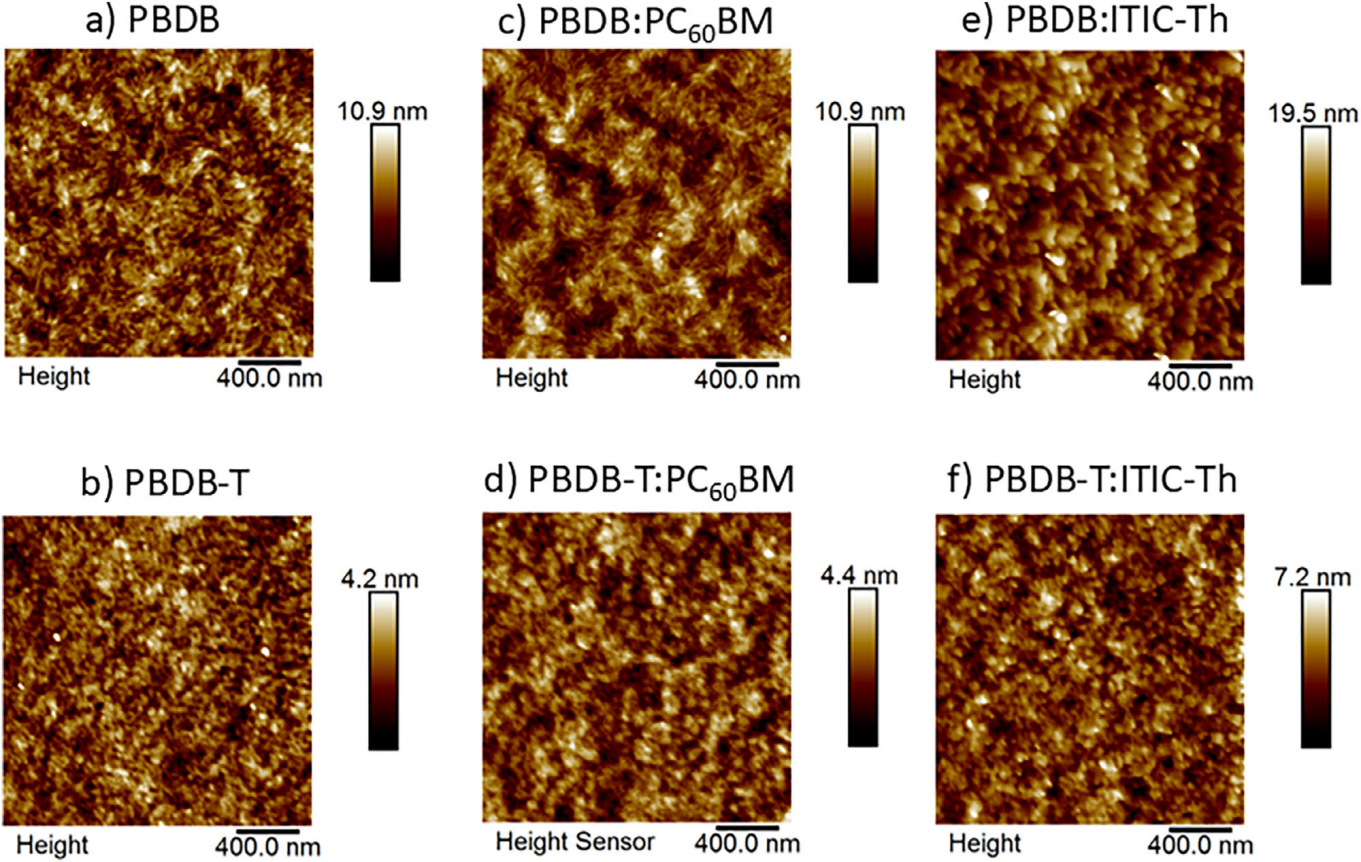
Atomic force microscopy images for (a) PBDB pristine (b) PBDB‐T pristine, (c) PBDB:PC60BM, (d) PBDB‐T:PC60BM (e) PBDB:ITIC‐Th, and (f) PBDB‐T:ITIC‐Th films. Blends have a weight ratio of 1:0.8.

Atomic force microscopy (AFM) images of the different films are shown in Figure 2 and the extracted data in Table 1. The two pristine polymer films exhibit quite different surface roughness levels, with PBDB showing an average roughness value of *R*_a_ = 1.2 nm, more than double that of PBDB‐T (*R*_a_ = 0.5 nm). The addition of fullerene leads to no significant changes in the surface roughness of the films compared to the pristine samples. This suggests that phase segregation is limited due to both polymers’ good miscibility with PC60BM. However, the PBDB:ITIC‐Th blend exhibits a large increase in roughness relative to the pristine sample, almost doubling. This may suggest stronger phase segregation relative to the fullerene blends, and that PBDB intermixes less with ITIC‐Th than with PC60BM. In contrast, PBDB‐T:ITIC‐Th shows only a small increase in surface roughness compared to the pristine polymer film, from 0.5 to 0.7 nm. The considerably smaller roughness increase in the PBDB‐T system may be indicative of better intermixing and the perhaps the presence of smaller domains compared to PBDB:ITIC‐Th (noting that AFM only reveals surface morphology). This is consistent with the PL showing more efficient exciton quenching in PBDB‐T, and implies that PBDB‐T:ITIC‐Th has a greater interfacial area.

**TABLE 1:** Average roughness, R_a_, and root mean square, R_q_, values extracted from AFM data.

Sample	PBDB		PBDB‐T	
	R_a_ (avg, nm)	R_q_ (RMS, nm)	R_a_ (avg, nm)	R_q_ (RMS, nm)
Pristine film	1.2	1.5	0.5	0.6
PC60BM 1:0.8 blend	1.1	1.4	0.5	0.6
ITIC‐Th 1:0.8 blend	2.2	2.8	0.7	0.9

**FIGURE 3:**
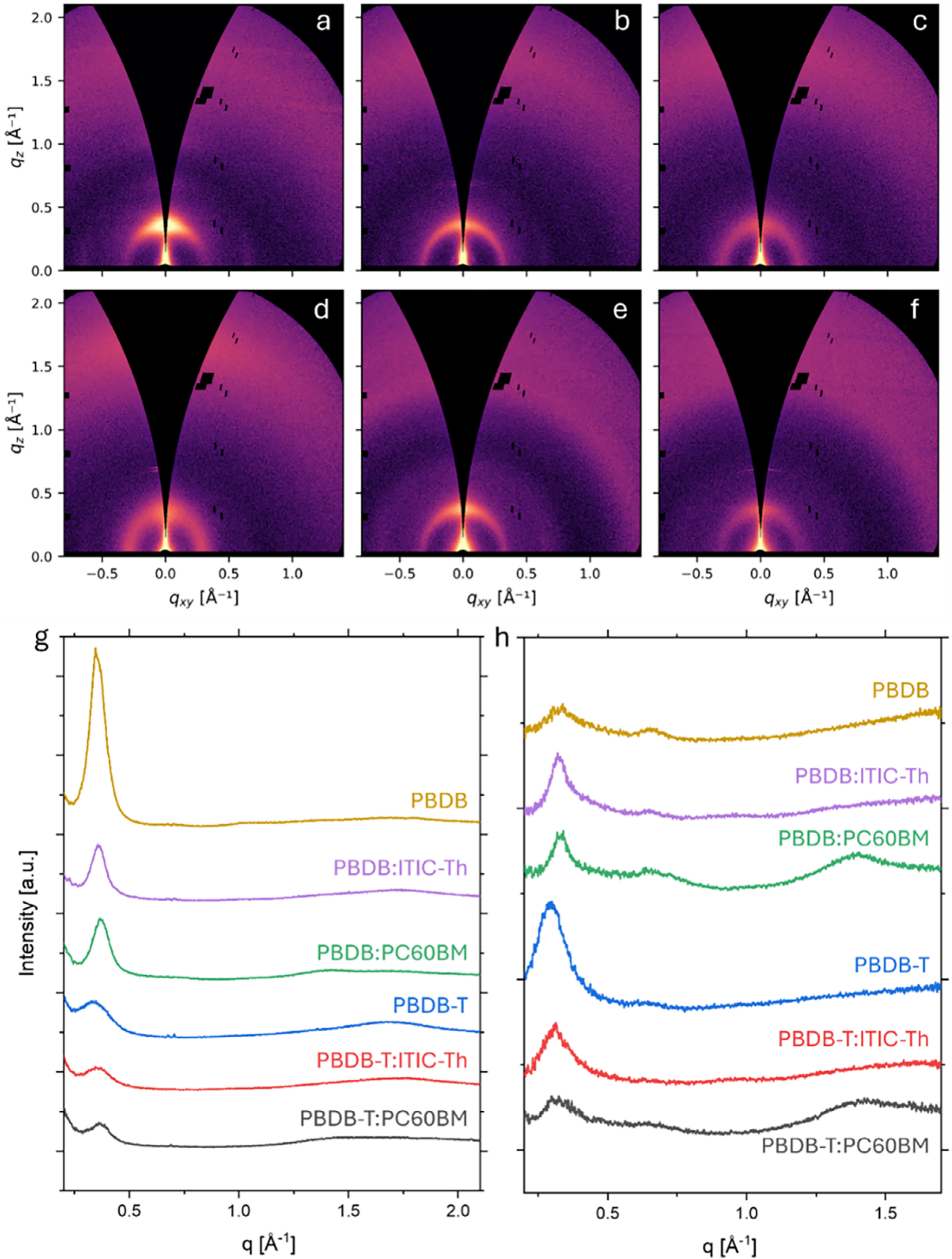
2D GIWAXS data of (a) PBDB, (b) PBDB:ITIC‐Th, (c) PBDB:PC60BM, (d) PBDB‐T, (e) PBDB‐T:ITIC‐Th and (f) PBDB‐T:PC60BM films on glass substrates. Corresponding 1D GIWAXS intensity profiles in the (g) out‐of‐plane (q_z_) and (h) in‐plane (q_xy_) directions. Data are offset by an arbitrary constant for clarity.

Grazing incidence wide‐angle X‐ray scattering (GIWAXS) was also performed to probe the molecular packing of the neat polymer and blend films. All films exhibit a pronounced scattering peak in the *q* range 0.30–0.37 Å^−^^1^, corresponding to the (100) lamellar stacking of the polymer [11]. Neat PBDB displays a strong lamellar peak at *q* = 0.353 Å^−^^1^ (*d* = 17.8 Å) with pronounced out‐of‐plane intensity, indicative of a predominantly edge‐on orientation (Figure 3). This is confirmed in *sin(χ)* corrected pole figures, which show considerably stronger intensity in the out‐of‐plane direction (*χ* = 0°) (Figure S3). Crystalline coherence lengths (*CCLs*) calculated via Scherrer analysis are considerably larger in the out‐of‐plane direction (8 nm) compared to the in‐plane direction (3.5 nm), consistent with long‐range lamellar stacking normal to the substrate (Table 2; Tables S1 and S2). Upon blending PBDB with ITIC‐Th and PC60BM, out‐of‐plane *CCLs* decrease slightly to 7.5 and 7.0 nm, respectively, while the in‐plane *CCLs* increase to 6.8 and 7.0 nm. In addition, *sin(χ)* corrected pole figures show a reduced population of edge‐on oriented polymer, suggesting that the out‐of‐plane lamellar ordering is significantly suppressed in the blends.

**TABLE 2:** GIWAXS data summary of d‐spacing and crystalline coherence lengths (CCL) corresponding to the in‐plane and out‐of‐plane lamellar peaks. Full tables are presented in the SI.

Sample	In‐plane *d*‐spacing [Å]	In‐plane *CCL* [nm]	Out‐of‐plane *d*‐spacing [Å]	Out‐of‐plane *CCL* [nm]
PBDB	18.64 ± 0.05	3.49 ± 0.06	17.78 ± 0.01	8.06 ± 0.06
PBDB:ITIC‐Th	19.09 ± 0.03	6.75 ± 0.11	17.59 ± 0.01	7.52 ± 0.06
PBDB:PC60BM	18.88 ± 0.03	7.03 ± 0.13	17.07 ± 0.01	6.97 ± 0.06
PBDB‐T	21.28 ± 0.02	4.98 ± 0.03	18.73 ± 0.02	3.25 ± 0.03
PBDB‐T:ITIC‐Th	20.00 ± 0.03	4.80 ± 0.06	17.92 ± 0.03	4.65 ± 0.05
PBDB‐T:PC60BM	19.41 ± 0.05	4.19 ± 0.07	17.22 ± 0.02	6.08 ± 0.06

In contrast, neat PBDB‐T exhibits reduced overall crystallinity compared to PBDB, as indicated by the presence of a considerably weaker intensity lamellar stacking peak at *q* = 0.335 Å^−^^1^ (*d* = 18.7 Å) and smaller *CCL*s of 3 nm (out‐of‐plane) and 5 nm (in‐plane) (Figure 1b). Interestingly, blending PBDB‐T with ITIC‐Th or PC60BM enhances the out‐of‐plane coherence to 4.7 and 6.0 nm, respectively, whilst only slightly reducing the in‐plane *CCL*s to 4.8 and 4.2 nm. This suggests that the acceptor components facilitate improved vertical packing in PBDB‐T‐based blends, whilst maintaining lateral lamellar ordering. In comparison to PBDB systems*, sin(χ)* corrected pole figures show that there is a considerably weaker azimuthal dependence in PBDB‐T based systems, consistent with a more isotropic molecular orientation (Figure S3).

Overall, these results demonstrate a more positive influence of the acceptor on the polymer packing in PBDB‐T compared to PBDB, with the PBDB‐T blends showing improved vertical packing and a higher degree of molecular orientation isotropy. In contrast, the GIWAXS results for the PBDB blends reveal more suppressed ordering.

### 2.3 Device Performance

Devices were constructed of the four blends using an ITO/PEDOT:PSS/active layer/PDINN/Ag structure. The results are shown in Table 3 and Figure S4. PBDB‐T device efficiencies reported in the literature vary anywhere from 2% up to 14% [7, 8, 10, 25, 26, 27, 28], depending on numerous factors which include acceptor identity, blend ratio, choice of processing additive, device structure and interlayers, and active layer thickness. For the unoptimized devices reported here, a maximum power conversion efficiency of 6.1% was obtained, but we note that this is in part because of the use of the simpler, conventional device structure rather than an inverted one, which typically achieves higher efficiencies for PBDB‐T [7]. This implies that the spectroscopic and morphology results are still likely to be representative of higher‐performing devices. In particular, the results are indeed consistent with the anecdotal evidence discussed in the introduction, where the polymers with alkylthienyl substituents have superior device performance compared to those with alkoxy substituents.

**TABLE 3:** The parameters measured for devices with an ITO/PEDOT:PSS/active layer/PDINN/Ag structure.

	Voc (V)	Jsc (mA.cm^−2^)	FF %	PCE %
PBDB:PC60BM	0.84	11.5	59.7	5.6
PBDB‐T:PC60BM	0.94	12.6	50.7	6.1
PBDB:ITIC‐Th	0.85	13.5	42.9	5.0
PBDB‐T:ITIC‐Th	0.92	15.9	40.9	6.0

Both PBDB‐T blends have higher power conversion efficiency (PCE) than the PBDB blends by 10%–20%. For both the NFA and fullerene blends, this improvement in performance was due to enhanced open‐circuit voltages (V_OC_) and short‐circuit currents (J_SC_), despite a reduced fill factor. The observation that these trends occurred with both types of acceptor indicate that it is the nature of the polymer that is driving the device characteristics. As such, it is important that we understand the role of polymer structure and its influence on photophysics and device performance better.

We note that the higher V_OC_ observed for the two PBDB‐T devices does not originate from the energetics of the polymer. The two polymers have similar optical bandgaps and similar HOMO levels, as measured by electronic spectroscopy and cyclic voltammetry respectively (Figure 1). This was confirmed by assessing the photovoltaic bandgap [29] of the devices (the derivative of the external quantum efficiency vs energy, Figure S4c), which provides the energetic difference between the HOMO of the donor and the LUMO of the acceptor. The data showed PBDB‐T:ITIC‐Th and PBDB:ITIC‐Th to have identical photovoltaic bandgaps. PBDB‐T:PC60BM and PBDB:PC60BM blends also have identical photovoltaic bandgaps, albeit ∼0.2 eV higher than the two ITIC‐Th blends. Since the acceptors are the same in each pairing, this indicates that the HOMOs of the two polymers are very similar, consistent with literature reports [30].

### 2.4 Transient Absorption Spectroscopy (TAS)

**FIGURE 4:**
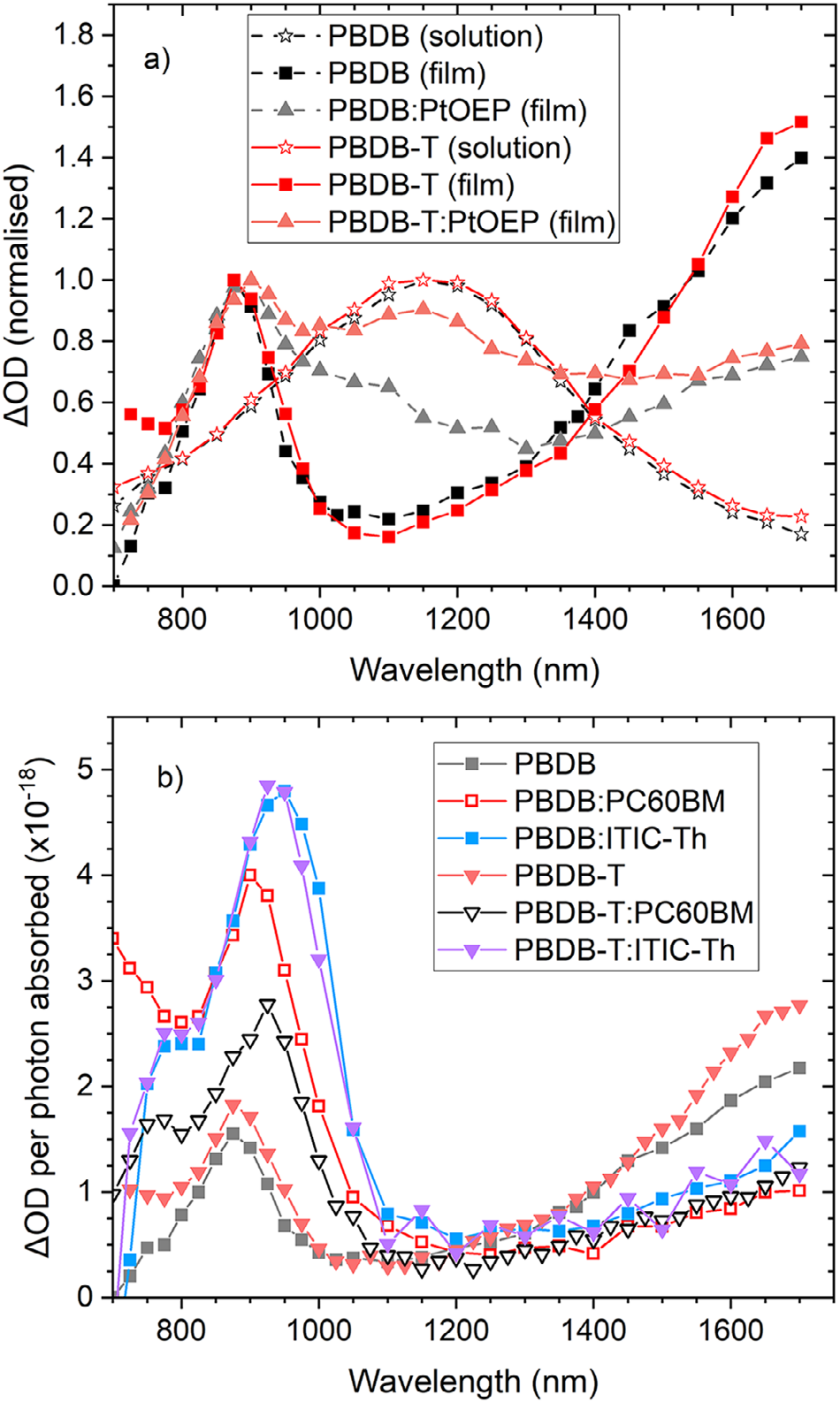
(a) Normalized TA spectra of the polymer pristine films, solutions (chlorobenzene), and PtOEP sensitization experiments at 1.5 µs, showing the presence of triplets in PBDB and PBDB‐T at 1150 nm. (b) TA spectra at 1.5 µs of the blend and pristine films, normalized per photon absorbed to assess relative polaron populations. All blend films were excited at 600 nm, 12 µJ cm^−2^. All blend weight ratios are 1:0.8. The solutions were excited at 600 nm, 12 µJ cm^−2^. The sensitized polymer:PtOEP (1:3) films were excited at 540 nm, coinciding with the PtOEP Q‐band.

We begin with µs‐TAS, as this can assist with the identification of longer‐lived transient species, and the spectra are typically simpler, owing to the absence of singlet states. Note that the triplet and radical anion spectra of ITIC‐Th have already been reported [31]. The ITIC‐Th triplet has previously been identified at 1150 nm, while the ITIC‐Th radical anion has been observed at 795 nm. As such, we focus on the pristine polymers. Both PBDB and PBDB‐T pristine film TA spectra are very similar (Figure 4a), with a band at 860 nm and a rising tail toward 1600 nm. These are very similar to the µs‐TA spectrum of PM6 previously reported [32], noting that PM6 is structurally similar to the two polymers discussed here. For the PM6, a detailed study as a function of acceptor ratio revealed that the >1600 nm band is related to bound charge carriers in ordered, homogeneous domains while the 860 nm band can be assigned to more interfacial charge carriers. Given the structural similarities between PM6, PBDB, and PBDB‐T, it is likely that the same assignment applies here.

Since triplet states are known to occur in blends with NFAs, the TA spectra of the polymer triplet states were also acquired for both solutions and sensitized films (Figure 4a). Both PBDB and PBDB‐T solutions in chlorobenzene reveal identical polymer triplet spectra, with a single broad band at 1160 nm that displays strong oxygen sensitivity, very similar to what has been previously reported for PM6 [33]. To check if the triplet band shifts in the solid phase, sensitized films with platinum octylethylporphyrin (PtOEP) were also measured. Selective excitation was not possible, so the measured TA spectra are a combination of what was observed for the pristine polymer and the contribution from the sensitized polymer triplet (the PtOEP triplet absorbs below 800 nm [34]). The spectral position of the polymer triplet in the sensitized film is in the same spectral position as in solution. To check if any triplets are apparent in the pristine polymer films, oxygen sensitivity experiments were performed (Figure S5). These showed a weak oxygen sensitivity, with the full signal recovered when the inert atmosphere was replaced. Since no clear triplet band is observed in Figure 4b, this result implies a small population of triplets underlying the charge carrier absorption bands.

The µs‐TA data for the four blend films are shown in Figure 4b. In all cases, the relative amplitude of the >1600 nm band decreases from the pristine to the blend, as was also observed for the PM6 blends previously reported [32], and this is consistent with a reduction in the proportion of pristine polymer domains in the blend film. As such, we use the 860 nm band as an indicator of relative charge carrier population, noting the likely presence of a small triplet contribution at this wavelength. As expected from the efficient exciton quenching observed in the PL, the blend films show greater charge carrier populations compared to the pristine polymers. Interestingly, the two ITIC‐Th blends show identical TA spectra, both in terms of spectral position (shifted to 940 nm) and amplitude, indicating that the charge carrier population on microsecond timescales is very similar. For the fullerene blends however, the PBDB:PC60BM shows a larger charge carrier population at 1.5 µs than PBDB‐T:PCBM, which is partly caused by slightly slower decay kinetics (*t*_1/2_ = 1.7 and 1.3 µs, respectively, Figure S6).

**FIGURE 5:**
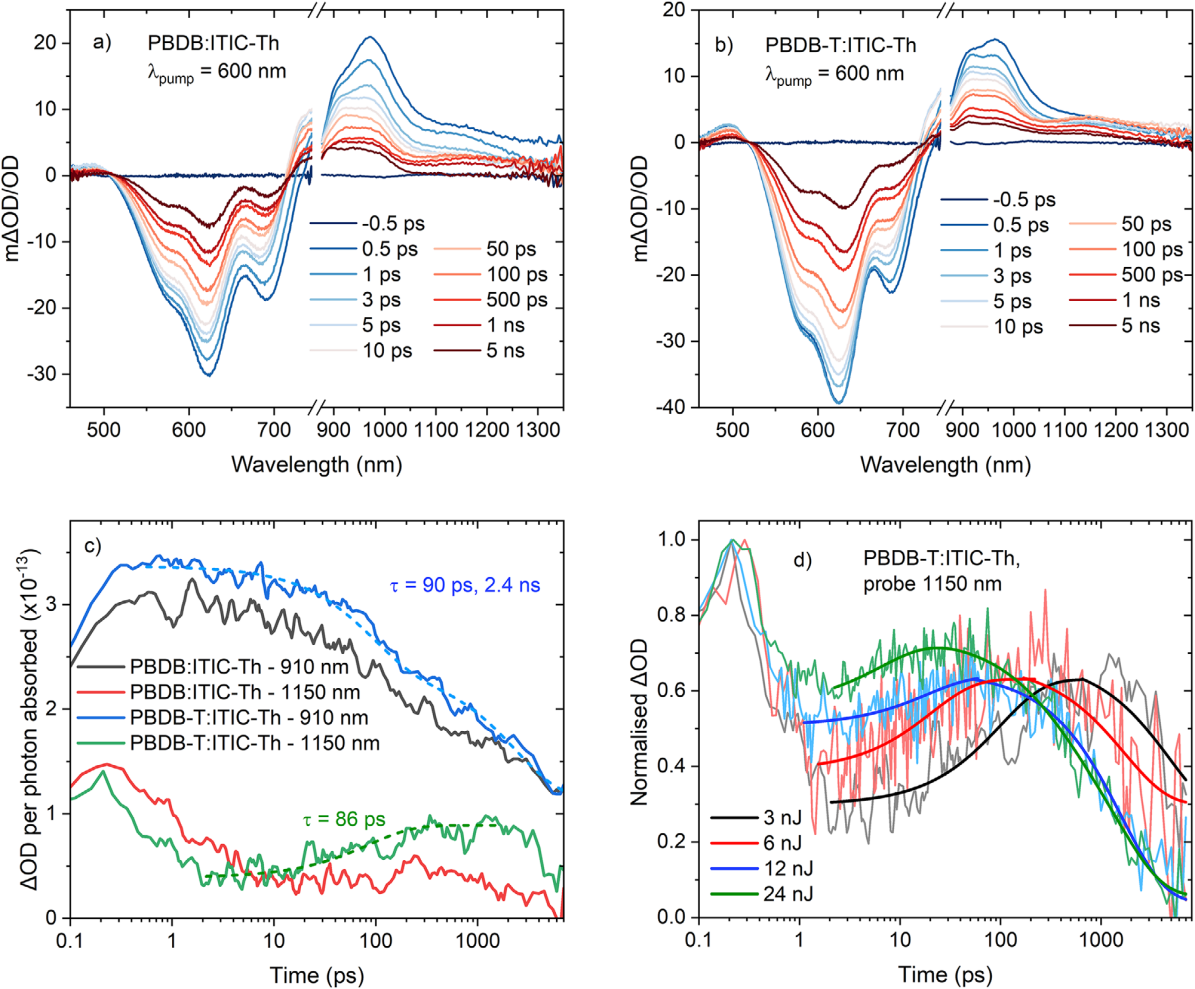
Ultrafast TA spectra of (a) PBDB:ITIC‐Th and (b) PBDB‐T:ITIC‐Th films, using 12 nJ excitation. (c) Raw kinetics of the two ITIC‐Th blends at 910 nm (polymer polaron; probed at 910 nm to minimize spectral contamination from the ITIC‐Th exciton at 980 nm) and 1150 nm (polymer singlet exciton and NFA triplet state), corrected for photons absorbed, using 3 nJ excitation. (d) The kinetics for the PBDB‐T:ITIC‐Th film probed at 1150 nm as a function of fluence. The solid lines are guides for the eye. All data in this figure was measured using a pump wavelength of 600 nm.

Now that the spectral positions of the polymer charge carriers and triplets have been identified, in addition to some preliminary insights into charge carrier populations on long times, we turn to ultrafast TAS to extend the understanding of the photophysical mechanisms in play. The pristine materials’ TA data are shown in Figure S7, the fullerene blends in Figure S8, and the ITIC‐Th blends in Figure 5. An excitation wavelength of 600 nm was chosen, close to the polymer absorption maximum but exciting both donor and acceptor. The ground state bleach (GSB) of the ITIC‐Th blends clearly contain contributions from both the polymer (620 nm) and the ITIC‐Th (690 nm). As seen from the pristine polymer data, the singlet exciton for both polymers is very broad and encompasses the majority of the NIR spectral region. In the ITIC‐Th blends, the polymer singlet exciton is largely quenched. However, the ITIC‐Th singlet exciton is observed at early times at ∼980 nm, alongside the longer‐lived charge carriers at 940 nm. The more prominent appearance of the ITIC‐Th singlet exciton compared to the polymer singlet may be indicative of ultrafast energy transfer, as has been observed in numerous polymer:NFA blends previously [16, 17, 32].

An additional observation in the polymer:ITIC‐Th blends is the appearance of a new TA band at 1150 nm at longer times (Figure 5a,b). A comparison of this 1150 nm band to the µs‐TA spectra of both polymer and ITIC‐Th triplets shows that the FWHM of the new band is more consistent with the ITIC‐Th triplet (Figure S9). In Figure 5c, a closer examination of the 1150 nm kinetics using the lowest excitation energy of 3 nJ (see Figure S10a) reveals that the rise of this triplet (τ = 86 ps) matches one of the time components of the decay of the charges at 940 nm (90 ps), indicating that the triplets are forming via charge recombination. The growth of the triplet absorption is strongly fluence dependent, denoting a non‐geminate triplet formation mechanism (Figure 5d) [35, 36]. Interestingly, it is observed that the triplet population in the PBDB‐T:ITIC‐Th film is greater than in the PBDB:ITIC‐Th film, and this is consistent with the larger charge photogeneration yield observed at 910 nm for the PBDB‐T:ITIC‐Th film on ps‐ns timescales. More charges enable more recombination, which results in a greater triplet population. However, it was also noted that while the ITIC‐Th triplet population is doubled in PBDB‐T:ITIC‐Th compared to PBDB:ITIC‐Th, the polymer polaron population at early times is only ∼40% larger. This is partially due to the slightly slower polaron decay observed for PBDB:ITIC‐Th (which has a decay component of 95 ps). This leads to both a smaller maximum triplet population in PBDB:ITIC‐Th and to the two blends having similar polaron populations at later times. This slower polaron decay is consistent with the slightly higher FF and stronger phase segregation observed for PBDB:ITIC‐Th. However, the smaller FF is overshadowed by the larger J_SC_ and V_OC_ seen for PBDB‐T:ITIC‐Th, which may suggest that triplet formation does not inhibit device performance as strongly in PBDB‐T:ITIC‐Th as has been observed in other systems [36].

To explore in more detail why the PBDB‐T:ITIC‐Th film creates more charge carriers than PBDB:ITIC‐Th, the influence of the ITIC‐Th acceptor must be considered. The ultrafast TA spectra of the two ITIC‐Th blends (Figure 5a,b) reveal a smaller ITIC‐Th singlet exciton amplitude at 980 nm in the early (0.5–3 ps) time regime for the PBDB‐T:ITIC‐Th film. This could be due to either less efficient energy transfer from the polymer in the case of the PBDB‐T, or more efficient hole transfer from the ITIC‐Th singlet exciton [21, 37, 38, 39]. It is unlikely that the energy transfer from the PBDB‐T is less efficient due to the very similar energetics in both polymers. Furthermore, PBDB‐T has a slighter greater fluorescence quantum yield than PBDB (Figure S2), and this would enhance FRET‐mediated energy transfer to the ITIC‐Th, not reduce it. Indeed, a comparison of the polymer singlet exciton kinetics at early times (probe 1150 nm, Figure 5c) indicates that PBDB‐T:ITIC‐Th has faster exciton quenching than PBDB:ITIC‐Th, and this is consistent with more efficient energy transfer, not less. Singlet energy transfer from the ITIC‐Th to the polymer must also be considered, but this is highly unlikely in this case due to ITIC‐Th's lower S_1_ energy, minimal overlap between the ITIC‐Th emission and polymer absorption, and the small fluorescence quantum yield of ITIC‐Th of ∼1% [28]. As such, the reduced presence of the ITIC‐Th singlet exciton in the PBDB‐T:ITIC‐Th film spectra is more likely to be a higher efficiency of hole transfer, and this is indeed consistent with the greater charge photogeneration yield observed for this blend.

**FIGURE 6:**
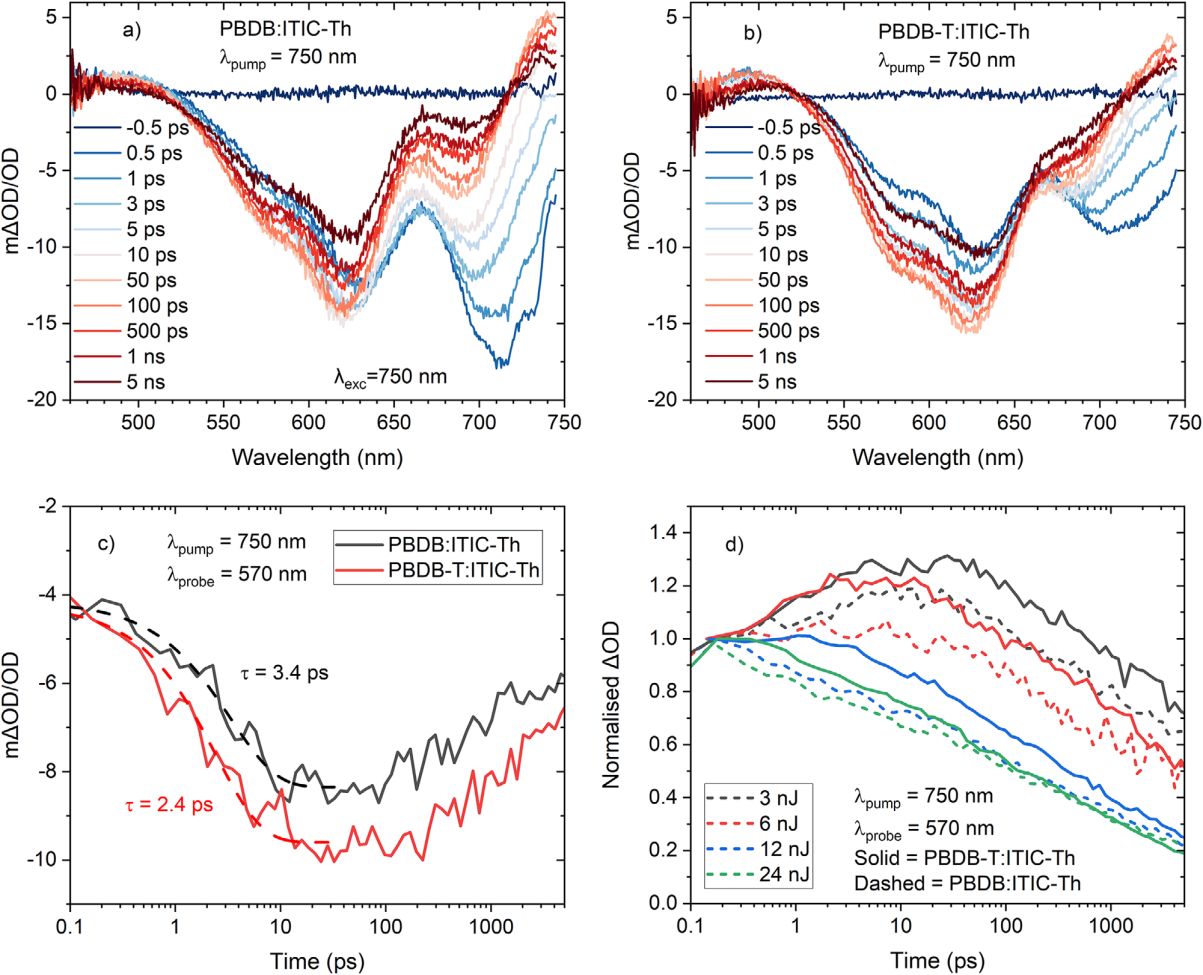
Ultrafast TA spectra of (a) PBDB:ITIC‐Th and (b) PBDB‐T:ITIC‐Th films, and using 12 nJ excitation. (c) Raw kinetics of the two ITIC‐Th blends at 570 nm, representing the polymer ground state bleach, using 3 nJ excitation. (d) The kinetics for the two ITIC‐Th blends probed at 570 nm as a function of fluence. All data in this figure were measured using a pump wavelength of 750 nm (selective ITIC‐Th excitation).

To confirm the hypothesis of more efficient hole transfer in the PBDB‐T:ITIC‐Th film, we repeated the ultrafast TAS experiments with an ITIC‐Th selective excitation wavelength of 750 nm. This allows for the hole transfer process to be directly measured by assessing the growth of the polymer GSB. The data are shown in Figure 6. Even at the earliest time of 0.1 ps, polymer GSB is apparent for both polymer blends, indicating hole transfer is occurring efficiently in both cases, and that a certain percentage of the transfer occurs prior to the time resolution of the instrumentation [37, 38, 39]. Despite this, it can also be observed that the relative proportion of polymer GSB to ITIC‐Th GSB in the PBDB‐T:ITIC‐Th film is substantially higher compared to the PBDB:ITIC‐Th film, which is strongly indicative of more efficient hole transfer in PBDB‐T:ITIC‐Th (Figure 6a,b). This is also evident from a comparison of the kinetics at the polymer GSB (570 nm, Figure 6c). 570 nm is chosen as the probe wavelength to minimize contributions from the adjacent ITIC‐Th GSB whilst still maintaining good polymer GSB signal amplitude. The polymer GSB grows in immediately from very early times for PBDB‐T:ITIC‐Th, with a rise time of 2.4 ps and reaches a greater amplitude. In contrast, the PBDB:ITIC‐Th film shows a slower hole transfer with a rise time of 3.4 ps and does not reach the same amplitude. These data are indeed consistent with a greater hole transfer efficiency for PBDB‐T:ITIC‐Th compared to PBDB:ITIC‐Th. This contributes to the higher charge photogeneration efficiency observed for PBDB‐T:ITIC‐Th film, as assessed from the polymer polaron population at 910 nm (Figure 5).

An additional contributing factor to the improved charge photogeneration efficiency in PBDB‐T:ITIC‐Th is likely the longer polymer singlet state lifetime observed for pristine PBDB‐T (t_1/2_ = 50 ps) compared to PBDB (t_1/2_ = 30 ps) under 3 nJ excitation, shown in Figure S10b. Fluence‐dependent data reveal that 3 and 6 nJ produce almost identical kinetics for the pristine films, suggesting that intrinsic behavior has been reached (Figure S10a). However, even at 3 nJ, the kinetics can only be fitted by a biexponential function, indicating that it is possible that aggregation in the condensed phase is leading to some degree of excited‐state quenching. Rapid decay kinetics for pristine PBDB‐T have indeed been previously reported [40, 41]. Despite this, the longer exciton lifetime for PBDB‐T compared to PBDB under these specific excitation conditions allows more time for beneficial processes that contribute to charge photogeneration—such as energy or electron transfer—to take place.

However, recalling the similar charge carrier yields on much longer µs timescales between the two ITIC‐Th blends, it is clear that the gain in charge carrier population from more efficient hole transfer in PBDB‐T:ITIC‐Th is then lost due to enhanced triplet formation. Indeed, in Figure 5c, we can see that already at 4 ns—past the peak in triplet population—the two ITIC‐Th blends have identical charge carrier populations (910 nm). Nevertheless, the difference in hole transfer efficiency between such structurally similar systems is very interesting, and thus we turn to Raman spectroscopy to generate links between structure and electronic properties.

### 2.5 Raman Spectroscopy

**FIGURE 7:**
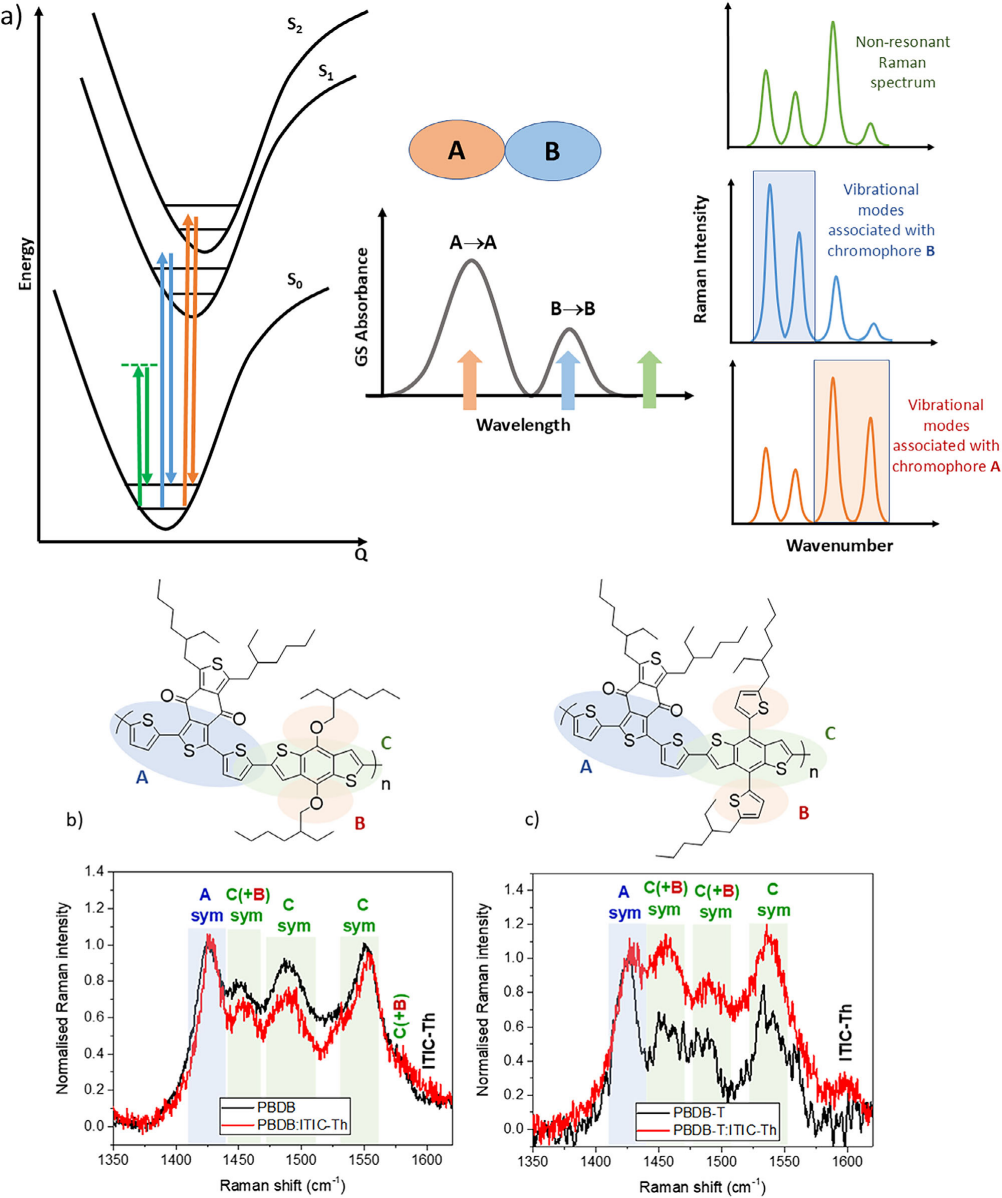
(a) Simplified schematic example showing the basics of resonance Raman spectroscopy for a generic dual‐chromophore molecule with localized π‐π* transitions on each chromophore. Green denotes non‐resonant conditions, and blue and orange denote resonant conditions with a B‐centered transition to S_1_ and an A‐centered transition to S_2_, respectively. The resonance Raman spectra and assignments for (b) pristine PBDB and PBDB:ITIC‐Th and (c) pristine PBDB‐T and PBDB‐T:ITIC‐Th, using an excitation wavelength of 488 nm and a power of 0.2 mW. “Sym” refers to a symmetrical stretching mode, noting that this type of mode is the one most likely to incur resonance intensity enhancements. The colour codes in (b) and (c) are as follows: blue “A” refers to a BDD‐centred mode, red "B" refers to a peripheral mode, and green “C” refers to a BDT‐centred mode.

As a scattering technique that provides vibrational information, Raman spectroscopy can enable insight into structure, bonding, and conformation [12]. The Raman intensity of a band is related to the polarizability of the electrons associated with that normal mode, and this makes Raman spectroscopy ideal for investigating π‐conjugated systems. An additional huge advantage of Raman spectroscopy is the resonance Raman effect: a selective enhancement of specific vibrational modes that are coupled to the resonant electronic transition. Qualitatively, the vibrational modes that are enhanced are those that mimic the structural changes occurring during the coupled electronic transition. In essence, the most enhanced Raman bands for a particular excitation wavelength indicate which part of the molecule is most affected by the resonant electronic transition (Figure 7a). It can thus be used to establish the nature of the resonant electronic state [42].

Since the Raman spectra of large polyatomic molecules are often complex, computational chemistry on monomer and dimer models was used to analyse the normal modes for PBDB and PBDB‐T. The calculated Raman spectra (B3LYP/6‐31G(d)) of dimer models are compared to the FT‐Raman spectrum (absence of resonance conditions) in Figure S11. Different mode localizations are identified, allowing the observed bands to be assigned (see Figures S12 and S13 and Tables S3 and S4). In all assignments, the benzodithiophene‐dione (BDD) unit is denoted as “A” (Figure 7), the benzodithiophene (BDT) unit denoted as “C”, and the alkoxy/alkylthienyl group as “B”. Most notably, the strongest band measured at 1425 cm^−1^ for PBDB‐T and 1426 cm^−1^ for PBDB can assigned to the symmetric stretch of the BDD unit's thiophene rings common to both polymers. For PBDB‐T, the band measured at 1464 cm^−1^ and those between 1500 and 1600 cm^−1^ are modes primarily localized on the benzodithiophene (BDT) unit, while the 1489 cm^−1^ band has a large contribution from the peripheral thiophene rings. For PBDB, the bands measured between 1450 and 1560 cm^−1^ are BDT‐localized and the 1582 cm^−1^ band has a particularly strong contribution from the peripheral ether linkages.

An excitation wavelength of 488 nm has been used to (mostly) selectively excite the polymer, noting that ITIC‐Th has a distinct absorption minimum at this wavelength and thus will contribute substantially less to the blend resonance Raman spectra. This effect can be observed in Figure S14, where an excitation wavelength of 676 nm is resonant with the ITIC‐Th, and we can see its contributions to the Raman spectrum. As the excitation wavelength is decreased, large changes in the intensity pattern are observed. By 532 nm, the ITIC‐Th contributions have almost vanished, leaving only polymer vibrational modes.

The resonance Raman results using an excitation wavelength of 488 nm are shown in Figure 7b,c for PBDB and PBDB‐T and their blends with ITIC‐Th, normalized to the 1425 cm^−1^ band. There is a striking difference between the two polymers when the ITIC‐Th is added. In the case of PBDB, the changes are relatively minor, with the relative intensity between the two strongest bands at 1425 cm^−1^ (BDD‐localized) and 1552 cm^−1^ (BDT‐localized) barely changing in the presence of the ITIC‐Th. For PBDB‐T, however, very substantial changes are observed in the polymer Raman spectrum when the ITIC‐Th is added. All of the BDT modes become significantly more enhanced compared to the BDD mode at 1425 cm^−1^. This observation suggests that the nature of the underlying PBDB‐T electronic state has changed in the presence of the ITIC‐Th, such that it becomes more BDT‐centered. Despite 488 nm being on the edge of the main electronic absorption band, the resonant transition is still primarily the S_1_ state, as the same Raman intensity trends with the longer excitation wavelength of 532 nm are observed (Figure S15). Note that longer wavelength comparisons around the polymer absorption maximum (∼600 nm) have been specifically not considered due to the ITIC‐Th coming into resonance at these wavelengths, leading to unavoidable ITIC‐Th contributions to Raman activity in the region of interest (Figure S14). As such, to inspect only polymer‐based vibrational modes, we restrict the comparisons to bluer excitation wavelengths.

The substantially stronger enhancement of the BDT modes in PBDB‐T:ITIC‐Th (compared to the BDD mode) suggests that the BDT moiety is more strongly coupled to the S_0_ → S_1_ electronic transition of PBDB‐T in the blend, while the BDD moiety is now less coupled. This observation implies that the frontier molecular orbitals—and thus electron density—involved in the S_1_ state have redistributed and are localized more strongly on the BDT unit. The observation that this occurs in the presence of the ITIC‐Th indicates a stronger intermolecular interaction between polymer and NFA for PBDB‐T compared to PBDB. This is consistent with the GIWAXS data showing improved packing and more isotropic molecular orientations in PBDB‐T blends compared to PBDB blends. A stronger polymer/acceptor interaction is also consistent with the enhanced exciton quenching observed in the PL data for PBDB‐T:ITIC‐Th compared to PBDB:ITIC‐Th. Indeed, localization of molecular orbital density due to the influence of an adjacent acceptor has been reported previously [43]. It appears that ITIC‐Th influences the packing in any bulk phase of PBDB‐T that is present, similar to the acceptor‐induced ordering seen in previous systems [14, 15].

This stronger localization of the S_1_ state onto the PBDB‐T's BDT unit in the presence of the ITIC‐Th is likely facilitated by the extended conjugation to the peripheral thiophene linkers, which have been reported to enhance intermolecular π‐π interactions [44] (including between the ITIC‐Th and PBDB‐T). Indeed, an examination of the frontier molecular orbitals of the PBDB‐T monomer and dimer, as calculated using density functional theory, reveals some HOMO density on the peripheral rings, consistent with the extended conjugation (Figure S16). Further calculations on polymer π‐dimers (Figure S17) showed that both systems exhibit typical π–π stacked arrangements stabilized by non‐covalent van der Waals forces. The calculated π–π stacking energy for the PBDB dimer is −0.70 eV, with an interplanar separation of approximately 4.05 Å between adjacent monomeric units. In contrast, PBDB‐T exhibits a stronger interchain interaction, with an increased stacking energy of −0.85 eV and a reduced intermonomer distance of 3.95 Å. This enhancement is attributed to the improved planarity of PBDB‐T, which facilitates more effective π‐orbital overlap and thereby strengthens non‐covalent binding. These results demonstrate PBDB‐T's ability to form close interactions with neighboring π‐systems, as also evidenced by the resonance Raman results.

The dimer models (Figure S17) also showed that, while most LUMO density is primarily localized on the BDD unit (as expected), the PBDB‐T dimer has weak but distinct LUMO density on the BDT unit as well. Since the S_1_ state is primarily a HOMO‐LUMO transition, this observation is consistent with the stronger localization of the S_1_ state on the BDT unit when intermolecular interactions are present—as observed in the PBDB‐T:ITIC‐Th Raman results.

The enhanced localization of the S_1_ state on the BDT unit in the PBDB‐T:ITIC‐Th film can be correlated with the more efficient hole transfer observed in PBDB‐T:ITIC‐Th (compared to PBDB:ITIC‐Th). The localization promotes the π‐π interaction between polymer and NFA [44] such that the hole transfer is more facile. We also note that the enhanced intermolecular interactions observed in PBDB‐T:ITIC‐Th may also promote short‐range energy transfer from polymer to acceptor, as has been reported recently for PBDB‐T blends, and this is consistent with the faster polymer exciton quenching in PBDB‐T:ITIC‐Th [37]. As such, the enhanced charge photogeneration yield observed for PBDB‐T:ITIC‐Th is largely caused by the redistribution of π‐electron density onto the BDT promoting hole transfer (and possible hole transfer as well), as enabled by the improved interaction between polymer donor and acceptor. This provides a valuable structure‐function relationship for future molecular design.

## 3 Conclusions

As the organic photovoltaics community progresses toward the exclusive use of peripheral alkylthienyl‐substituted conjugated polymers rather than alkoxy‐substituted ones, this paper explores the mechanistic origins of this shift. Transient absorption spectroscopy across multiple timescales, atomic force microscopy, GIWAXS, and resonance Raman spectroscopy have been used to gain insight into this observation. The TA data reveal more efficient hole transfer in the case of PBDB‐T:ITIC‐Th compared to the alkoxy‐substituted PBDB. Furthermore, a greater triplet population—formed by charge carrier recombination—is observed for PBDB‐T:ITIC‐Th compared to PBDB:ITIC‐Th. As such, although PBDB‐T:ITIC‐Th exhibits a higher charge photogeneration yield at early times, the triplet formation results in a similar charge carrier population for the two blends on the microsecond timescales relevant for charge extraction in organic photovoltaic devices.

The more efficient hole transfer in PBDB‐T:ITIC‐Th compared to PBDB:ITIC‐Th can be linked to the structural and morphological differences of the polymers. The resonance Raman spectra of the NFA blends compared to the pristine polymers reveal a stronger coupling of the polymer's S_1_ state to the benzodithiophene (BDT) unit in PBDB‐T:ITIC‐Th, facilitated by the extra conjugation provided by the peripheral alkylthienyl groups. This may enable a stronger donor‐acceptor π‐π interaction between the PBDB‐T and ITIC‐Th (which shows improved molecular packing) and thus promotes hole transfer. These results demonstrate the mechanistic origin of a valuable structure‐function relationship for future molecular design.

## Conflicts of Interest

The authors declare no conflicts of interest.

## Supporting information




**Supporting File**: advs74013‐sup‐0001‐SuppMat.docx.

## Data Availability

The data that support the findings of this study are available from the corresponding author upon reasonable request.
