# Supplementary material for: Alkylthienyl Side Groups in Conjugated Polymers Enable Localization of π Electron Density and Facilitate Efficient Hole Transfer
Source: Adv Sci (Weinh). 2026 Jan 26;13(19):e16088. doi: 10.1002/advs.202516088 (PMC13045324; doi:10.1002/advs.202516088)
Supplement: Supplementary file 1 — Supporting File: advs74013‐sup‐0001‐SuppMat.docx. [file ADVS-13-e16088-s001.docx]

**SUPPORTING INFORATION**

**Alkylthienyl side groups in conjugated polymers enable localisation of π electron density and facilitate efficient hole transfer**

Hristo Ivov Gonev,^a^ Daniel Congrave,^b^ Junjun Guo,^a,c^ Jose Marin-Beloqui,^a,d^ Max Allen,^e^ David Bacon,^a^ Ankita Kumari,^f^ Saeed Shadabroo,^g^ He Zhu,^b^ Dibyajyoti Ghosh,^f^ Rachel C. Kilbride,^h,i^ Safa Shoaee,^g^ Hugo Bronstein,*^,b^ Tracey M. Clarke*^,a^

1. Department of Chemistry, University College London, Christopher Ingold Building, London WC1H 0AJ, United Kingdom
2. Department of Chemistry, University of Cambridge, Lensfield Road, Cambridge CB2 1EW, United Kingdom
3. Industrial Catalysis Centre, Department of Chemical Engineering, Tsinghua University, China.
4. Department of Physical Chemistry, Faculty of Science, University of Malaga, 29071 Málaga, Spain
5. School of Mathematics and Physical Sciences, University of Sheffield, Dainton Building, Brook Hill, Sheffield, S3 7HF, United Kingdom.
6. Department of Materials Science and Engineering and Department of Chemistry, Indian Institute of Technology, Delhi Hauz Khas, New Delhi-110016, India
7. Optoelectronics of Disordered Semiconductors, Institute of Physics and Astronomy, University of Potsdam, Karl-Liebknecht-Strasse 24-25, 14476 Potsdam-Golm, Germany
8. Department of Physics, University of Warwick, Coventry, CV4 7AL, United Kingdom.
9. XMaS, The UK Materials Science Facility, European Synchrotron Radiation Facility, F-38043 Grenoble, France.

**Experimental**

***Materials and Sample Preparation***.

Polymer molecular weights were determined using gel permeation chromatography (GPC) in chlorobenzene at 80 °C vs. polystyrene standards. PC60BM (>99% purity) was purchased from Solenne, ITIC-Th from Ossila and PtOEP (95% purity) from Sigma Aldrich. Solutions were prepared via dissolving the materials in spectrophotometric grade chloroform (≥99.8% purity, containing 0.5-1.0% ethanol as stabiliser) from Sigma Aldrich and stirring overnight at room temperature in a glovebox with a N_2_ atmosphere. Concentrations used for solutions were 10 mg/mL for pristine polymers, 10 mg/mL total for polymer:PC60BM and polymer:ITIC-Th blend films at 1:0.8 weight ratio and 10 mg/mL total for polymer:PtOEP blends at 1:3, 1:1 and 3:1 weight ratios. The films were prepared via spin-coating for 60 s at 1000 RPM from solution. Glass substrates were cleaned via separately sonicating in solutions of acetone and isopropanol for 15 min each. Unless stated otherwise, measurements were carried out under an inert atmosphere, using either a continuous nitrogen flow or a Young’s tap cuvette.

***Ground State Absorption Spectroscopy***.

Ground state absorbance spectra were obtained with a PerkinElmer UV/Vis Lambda 365 spectrometer.

***Photoluminescence Spectroscopy***.

Steady-state emission spectra were obtained at room temperature with a Horiba FluoroMax-4 spectrofluorometer and corrected for instrument response at the excitation wavelength.

***Microsecond Transient Absorption Spectroscopy.***

A pump-probe micro-millisecond TA spectroscopy set-up was used to measure the TA spectra and kinetics. Laser pulses (repetition rate 10 Hz, pulse duration 6 ns) were generated by a Nd:YAG laser (Spectra Physics, INDI-40-10). Excitation wavelengths were selected by a versaScan L-532 OPO and the excitation density was set using neutral density filters, measured by a ES111C power meter (Thorlabs). The probe light was provided by a quartz tungsten halogen lamp (IL1, Bentham). Probe wavelength selectivity was achieved using bandpass filters and a Cornerstone 130 monochromator (Oriel Instrument) before the detector. The TA signals were recorded with Si and InGaAs photodiodes. The signal from the photodiodes was preamplified and sent to the main amplification system with an electronic filter (Costronic Electronics), which was connected to an oscilloscope (Tektronics, DPO4034 B) and PC.

***Picosecond Transient Absorption Spectroscopy****.*

A commercial pump-probe TA spectroscopy set-up (Helios Fire - Ultrafast systems) was used to measure the TA spectra and kinetics. A Ti:sapphire regenerative amplifier system (Coherent Astrella-HE-USP) was employed to generate laser pulses at a wavelength of 800 nm, a repetition rate of 1 kHz and a duration of 40 fs. Approximately 1 W of output power was coupled into an optical parametric amplifier (OPA; Coherent OPerA Solo) that was used to tune the pump wavelength on the sample. Neutral density filters were used at the output of the OPA to reduce the pulse energy to 3 - 24 nJ. The probe beam was directed onto a motorized delay stage, allowing a pump-probe delay of up to 7 ns, before being focused through a non-linear crystal to create a broadband white-light continuum. The crystal was chosen based on the desired probe wavelengths, with sapphire covering 500 to 720 nm and YAG used for the near-infrared (NIR). The pump beam was directed through an optical chopper set to 500 Hz, before being focused down and spatially overlapped with the probe on the sample. The pump spot was deliberately made larger than the probe to avoid unpumped signal being detected, while the relative polarisations of the pump and probe beam were set at the magic angle of 54.7° in order to suppress polarisation effects. The sample was kept in a sealed cuvette with nitrogen atmosphere.

***Device Fabrication.***

Photovoltaic devices were fabricated on ITO-coated glass substrates using a conventional architecture of ITO/PEDOT:PSS/active layer/PDINN/Ag. A 30 nm PEDOT:PSS layer was spin-coated in air and annealed at 150 °C for 15 min. The active layer (~100 nm) was prepared from a chloroform solution of donor and acceptor (1:0.8 weight ratio, 16 mg/mL). A 15 nm PDINN layer was then spin-coated under nitrogen. Finally, a 100 nm Ag electrode was thermally evaporated under a base pressure of ~1 × 10⁻⁷ mbar.

***Current Density-Voltage (J-V) Measurements.***

J-V characteristics were measured under nitrogen using a Newport Oriel Sol2A solar simulator (AM1.5G, calibrated with a Si reference cell) and a Keithley 2400 SourceMeter. Temperature was controlled during the measurements.

External quantum efficiency Measurements: EQE spectra were recorded using an SR 830 DSP lock-in amplifier under monochromatic light from a halogen lamp. The signal was normalized using calibrated Si (300–1100 nm) and Ge (1000–1400 nm) photodiodes.

***Computational chemistry***.

Density functional theory calculations were accomplished using the B3LYP level of theory and a 6-31G(d) basis set and the Gaussian G03 engine for the frequency calculations, with an applied frequency scale factor of 0.9613.

***GIWAXS.***

GIWAXS measurements were performed using a Xeuss 2.0 SAXS/WAXS X-ray scattering instrument (Xenocs) equipped with a Genix 3D Cu kα micro focus X-ray source (Xenocs). Using this system, a collimated X-ray beam with an energy of 8.041 keV was directed towards the sample at an incident angle of 0.16 ° (a value which is close to the critical angle of the neat materials). Scattered X-rays were detected by a vertically offset Pilatus 1M detector (Dectris) positioned 285 mm from the sample. The sample to detector distance was calibrated by measuring a silver behenate standard in transmission geometry and fitting the scattering rings in the pyFAI calibration GUI.^1^ Data was corrected, reduced and reshaped using batch processing scripts based on pyFAI and pygix libraries.^1^ 1D intensity profiles were produced by azimuthally integrating the 2D pattens across the full *q* range through various χ angles normal to the beam incidence at the detector; out-of-plane (*q_z_* direction in reciprocal space, -20 ° ≤ *χ* ≤ 20 °) and in-plane (*q_xy_* direction in reciprocal space, 65 ° ≤ *χ* ≤ 90 °). Sin(χ) corrected pole figures of the lamellar peak were produced by integrating across in the full *χ* range (0 ° ≤ *χ* ≤ 90 °) in the *q* range 0.3 Å^-1^ ≤ *q* ≤ 0.4 Å^-1^. The 100 lamellar stacking peaks in the out-of-plane and in-plane 1D profiles were fitted using a Lorentzian function and crystalline coherence lengths (*CCL*s) were estimated using the Scherrer equation: *CCL = 2πK/Δq* where *K* is the shape constant and *Δq* is the FWHM of the scattering peak. The shape constant can be a source of considerable debate and typically has values between 0.8 and 1.0. Here, a value of 1 is employed as the *CCL* values are only used to compare differences across the sample series.

***Synthesis.***

BDTT (CAS: 1352642-37-5) and BDD (CAS: 1415929-78-0) monomers were purchased from Ossila. DTOEHSn (CAS: 1160823-78-8) was synthesised according to the literature.^2^ The syntheses of PBDB and PBDB-T were inspired by a literature method.^3^

**Polymer synthesis.** Polymerisations were carried out on 100 mg scales of the corresponding bis(stannane). The bis(stannane) monomer (1.00 eq.), BDD (1.00 eq.) and Pd(PPh_3_)_4_ (4 mol.%) were combined in an oven and flame dried crimp cap 5 mL microwave vial under argon. Dry, degassed toluene (3 mL) was added and the resulting solution sparged with argon for 5 min before it was heated in a 120 °C oil bath for 20 h. The resulting dark reaction mixture was added dropwise into vigorously stirring methanol (200 mL). The precipitate was filtered into a Soxhlet thimble and then washed in a Soxhlet extractor with acetone until the eluent ran clear It was then washed with hexane until the eluent ran clear, and finally extracted with chlorobenzene. The solvent volume was reduced to *ca.* 5 mL and the residue added dropwise into vigorously stirring methanol (200 mL) to precipitate the desired polymers, which were isolated via filtration and washed copiously with acetone.

**PBDB** Synthesised from BDTOEHSn and BDD, yield 130 mg
GPC (chlorobenzene) Mn = 14,800, Mw = 52,600, PDI = 3.6.

**PBDB-T** Synthesised from BDTT and BDD, yield 130 mg
GPC (chlorobenzene) Mn = 22,400, Mw = 97,700, PDI = 4.4.

Polymerisations were carried out on a ca. 100 mg scale of the corresponding bis(stannane). The stannane monomer (1.00 eq.), palladium-5,15-di(4- bromophenyl)-2 2 ,23 ,72 ,73 ,122 ,123 ,172 ,173 -octamethyltetrabenzoporphyrin (0.00 eq. for 0 mol%, 0.10 eq. for 5 mol%), tri(ortho-tolyl)phosphine (0.12 eq.) and bis(dibenzylideneacetone)palladium(0) (0.03 eq.) were combined in an oven and flame dried crimp cap 5 mL microwave vial under argon. Dry, degassed chlorobenzene (3 mL) was added immediately, followed by 2,5-dibromothiophene (X) (0.90 for 5 mol%, 1.00 eq. for 0 mol%), and the resulting solution (orange/ red for blanks, dark green for porphyrin-doped) was degassed for 5 min. The vial was then heated in a microwave reactor sequentially at 100 °C for 2 min, 125 °C for 2 min and finally at 150 °C for 1 h. The resulting dark red reaction mixture was added dropwise into vigorously stirring methanol (200 mL). The precipitate was filtered into a Soxhlet thimble and then washed in a Soxhlet extractor with acetone until the eluent ran clear (note: while this may take ca. 2 h for the blank polymers, it was carried out overnight for porphyrin-doped polymers to ensure the removal of unreacted free porphyrin). It was then washed with hexane for ca. 3–5 h until the eluent ran clear, and finally extracted with chloroform. The solvent volume was reduced to ca. 5 mL and the residue added dropwise into vigorously stirring methanol (200 mL) to precipitate the desired polymers, which were isolated via filtration and washed copiously with acetone. C16 0% Pd. GPC (chlorobenzene) Mn = 29,000, Mw = 48,000, PDI = 1.7. C16 5% Pd. GPC (chlorobenzene) Mn = 14,000, Mw = 31,000, PDI = 2.2. C2C4 0% Pd. GPC (chlorobenzene) Mn = 10,000, Mw = 17,000 PDI = 1.7.

**Figure S1.** Comparison between the normalised absorbance (solid lines) and emission data (dashed lines) for the pristine polymer films as well as the PC60BM blend films. For the emission spectra, an excitation wavelength of 600 nm was used.

**Figure S2.** Photoluminescence spectra (corrected for absorbance) of the pristine polymers and their (a) fullerene and (b) non-fullerene blend films, showcasing the exciton quenching observed in blends. 98% quenching is seen for PBDB-T:PCBM and 82% for PBDB:PCBM, as calculated based on the areas under the curves. For the ITIC-Th blends, due to the emission contribution from both donors and acceptors, the quenching values (99% for PBDB-T:ITIC-Th and 97% for PBDB:ITIC-Th) are calculated based on the areas under the curves below 700 nm only, to exclusively account for the polymer contribution). The excitation wavelengths for all samples are 600 nm.

**Figure S3.** Sin(χ)corrected pole figures of the lamellar peak at q ~ 0.35 Å^-1^ for neat polymer and blend films.

**Table S1:** Peak position (q_peak_), d-spacing (d), FWHM (Δq) and crystalline coherence lengths (CCL) corresponding to the out-of-plane lamellar peak.

| **Sample** | **Out-of-plane lamellar peak, *q_peak_* [Å^-1^]** | ***d*-spacing (*d=2π/q_peak_*) [Å]** | **FWHM, *Δq* [Å^-1^]** | ***CCL* [nm]** |
| --- | --- | --- | --- | --- |
| PBDB | 0.353 ± 0.002 | 17.78 ± 0.01 | 0.078 ± 0.001 | 8.06 ± 0.06 |
| PBDB:ITIC-Th | 0.357 ± 0.002 | 17.59 ± 0.01 | 0.084 ± 0.001 | 7.52 ± 0.06 |
| PBDB:PC60BM | 0.368 ± 0.003 | 17.07 ± 0.01 | 0.090 ± 0.001 | 6.97 ± 0.06 |
| PBDB-T | 0.335 ± 0.004 | 18.73 ± 0.02 | 0.194 ± 0.002 | 3.25 ± 0.03 |
| PBDB-T:ITIC-Th | 0.351 ± 0.005 | 17.92 ± 0.03 | 0.135 ± 0.002 | 4.65 ± 0.05 |
| PBDB-T:PC60BM | 0.365 ± 0.004 | 17.22 ± 0.02 | 0.103 ± 0.001 | 6.08 ± 0.06 |

**Table S2:** Peak position (q_peak_), d-spacing (d), FWHM (Δq) and crystalline coherence lengths (CCL) corresponding to the in-plane lamellar peak.

| **Sample** | **In-plane lamellar peak, *q_peak_* [Å^-1^]** | ***d*-spacing (*d=2π/q_peak_*) [Å]** | **FWHM, *Δq* [Å^-1^]** | ***CCL* [nm]** |
| --- | --- | --- | --- | --- |
| PBDB | 0.337 ± 0.001 | 18.64 ± 0.05 | 0.180 ± 0.003 | 3.49 ± 0.06 |
| PBDB:ITIC-Th | 0.329 ± 0.001 | 19.09 ± 0.03 | 0.093 ± 0.002 | 6.75 ± 0.11 |
| PBDB:PC60BM | 0.333 ± 0.001 | 18.88 ± 0.03 | 0.089 ± 0.002 | 7.03 ± 0.13 |
| PBDB-T | 0.295 ± 0.001 | 21.28 ± 0.02 | 0.126 ± 0.001 | 4.98 ± 0.03 |
| PBDB-T:ITIC-Th | 0.314 ± 0.001 | 20.00 ± 0.03 | 0.131 ± 0.002 | 4.80 ± 0.06 |
| PBDB-T:PC60BM | 0.324 ± 0.001 | 19.41 ± 0.05 | 0.150 ± 0.003 | 4.19 ± 0.07 |


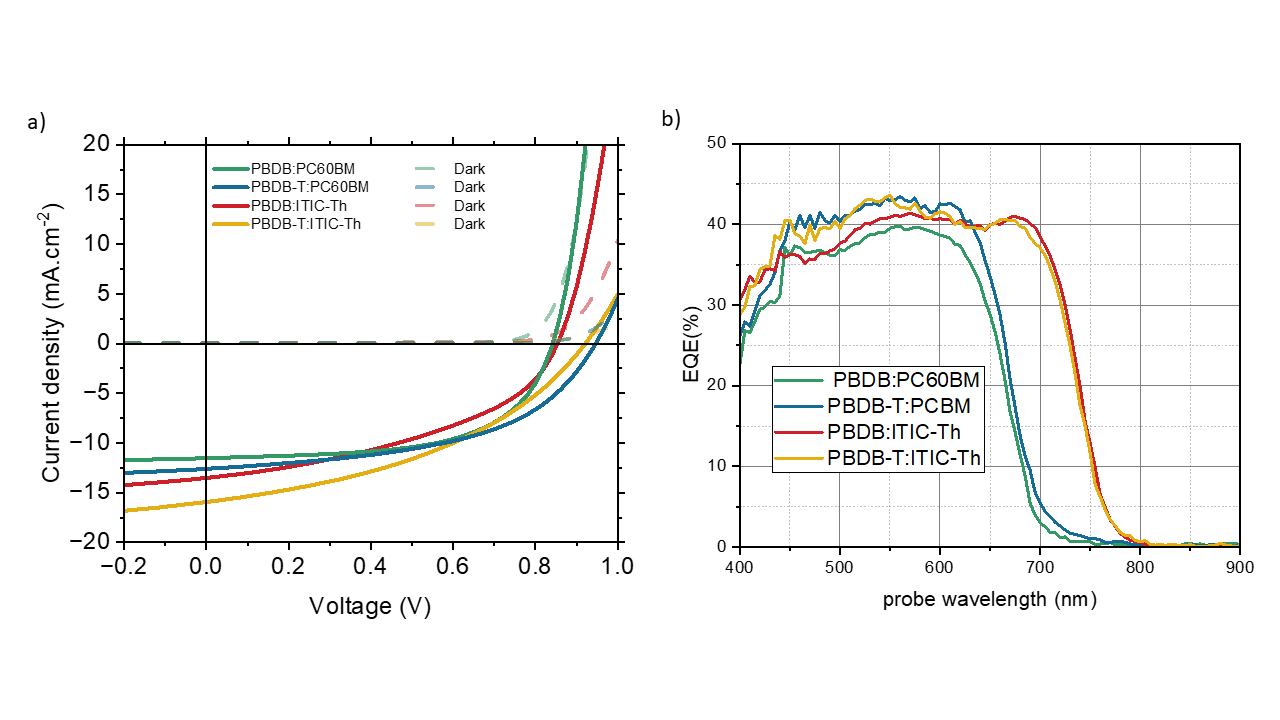


**
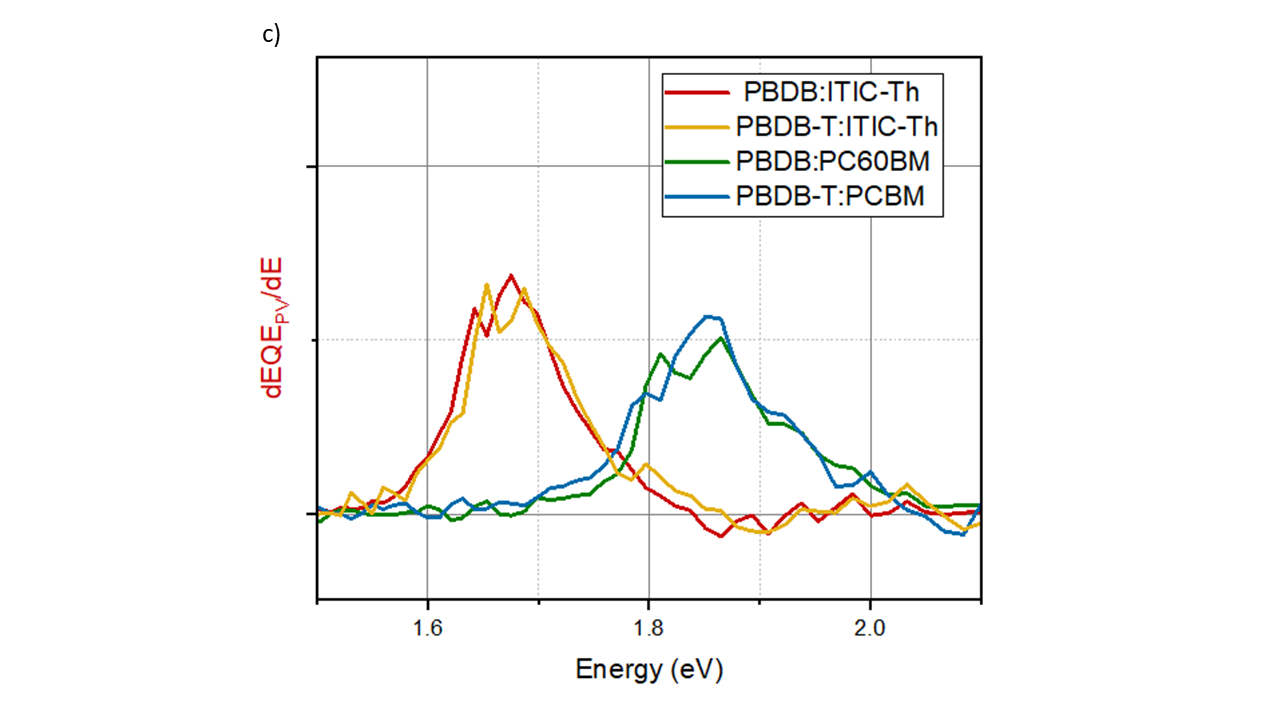
**

**Figure S4**. The (a) JV curves, (b) EQE spectra, and (c) photovoltaic bandgaps (taken from the derivative of the EQE with respect to energy) for the four blends with the device parameters reported in Table 3.

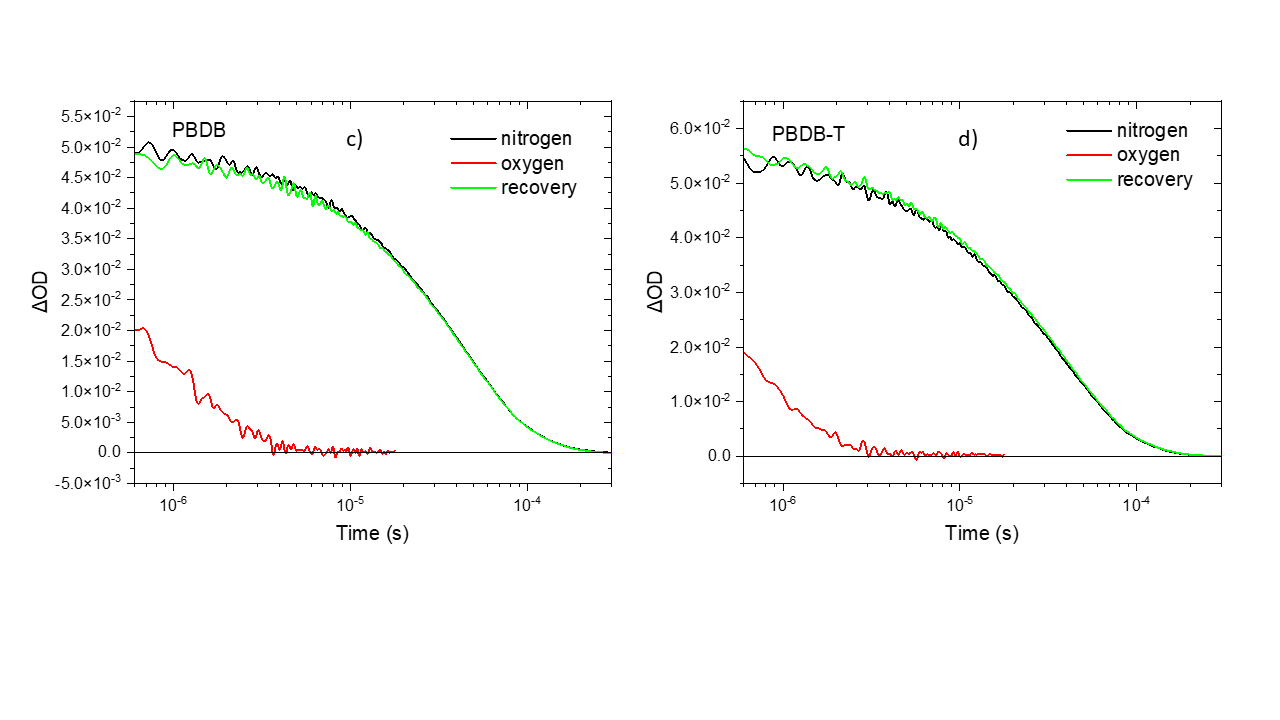


**Figure S5.** Weak oxygen dependence of the kinetics of pristine PBDB film, excited at 600 nm at 12 μJ cm^-2^ and probed at 900 nm (a) and 1600 nm (b). This suggests the presence of the broad triplet band at 1150 nm underlies the polaron bands observed at 900 nm and >1600 nm. This weak dependence can be compared to the very strong oxygen dependence of the triplets in solution in (c) and (d).

**Figure S6.** The μs-TA decay kinetics probed at 900 nm for the pristine polymers and (a) the PC60BM blends and (b) the ITIC-Th blends. All samples excited at 600 nm, 12 μJ cm^-2^. All blend weight ratios are 1:0.8.

**Figure S7.** PBDB, PBDB-T, and ITIC-Th pristine film ultrafast TA data – spectra taken at different delay times using 12 nJ excitation. Normalised ground state absorbance spectra have been added to the plots for reference. Excitation wavelength is 600 nm for the polymers and 750 nm for the ITIC-Th.

**Figure S8.** PBDB:PC60BM and PBDB-T:PC60BM blend film ultrafast TA data – spectra taken at different delay times using 12 nJ excitation. Excitation wavelength is 600 nm.

**Figure S9.** Normalised TA data for PBDB (a) and PBDB-T (b) systems, used to identify the nature of the triplet seen in 1:0.8 weight ratio polymer:ITIC-Th blend ultrafast TA data. The blend film spectra shown were measured at 24 nJ, 100 ps, to maximise the triplet signal. The pristine references (squares) are from the respective chlorobenzene solutions at 12 μJ cm^-2^, 1 μs. All samples excited at 600 nm.

**Figure S10.** (a) Singlet exciton decay kinetics for pristine PBDB-T film, excited at 600 nm and probed at 915 nm as a function of excitation energy. The 3 nJ and 6 nJ kinetics are very similar, suggesting that intrinsic behaviour has been reached. (b) The singlet exciton decay kinetics of the two pristine polymers, taken using the lowest fluence of 3 nJ and an excitation wavelength of 600 nm, showing the longer lifetime of the PBDB-T singlet compared to PBDB. The probe wavelength of 1050 nm avoids the polaron band at 900 nm and the triplet at 1150 nm as much as possible.


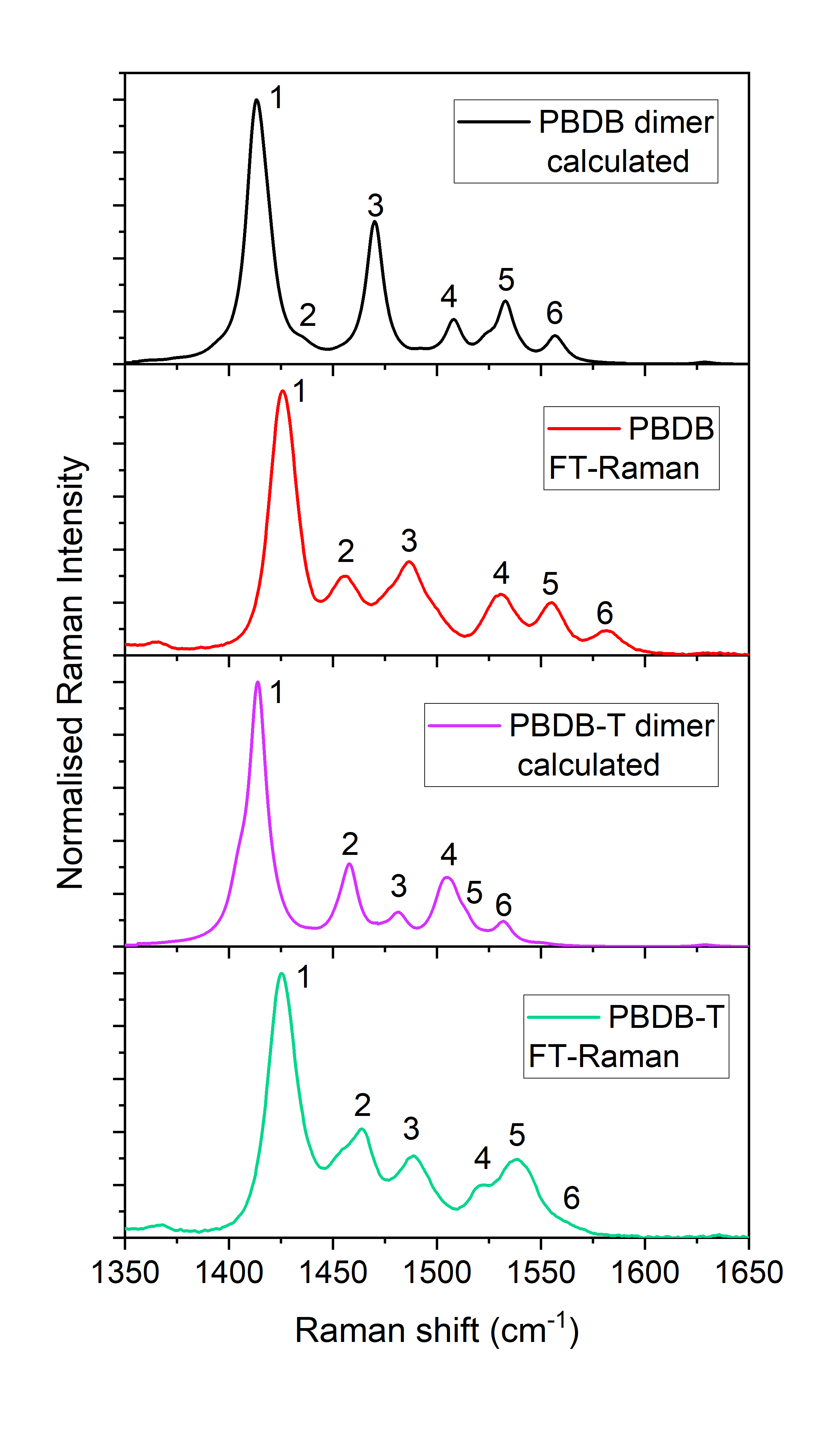


**Figure S11.** Calculated Raman spectra of the two polymers using dimer models (B3LYP/6-31G(d), frequency scale factor = 0.9613), compared to the measured FT-Raman spectra of powder samples (excitation wavelength = 1064 nm).

Raman assignments were made on the basis of Figure S11, comparing the non-resonant FT-Raman spectra to DFT-calculated dimer models of the two polymers. Frequency and intensity matching were employed, in addition to consideration of calculated monomer Raman spectra, and analysis of the resonance Raman spectra (noting that resonantly enhanced bands are most often symmetrical stretching modes).


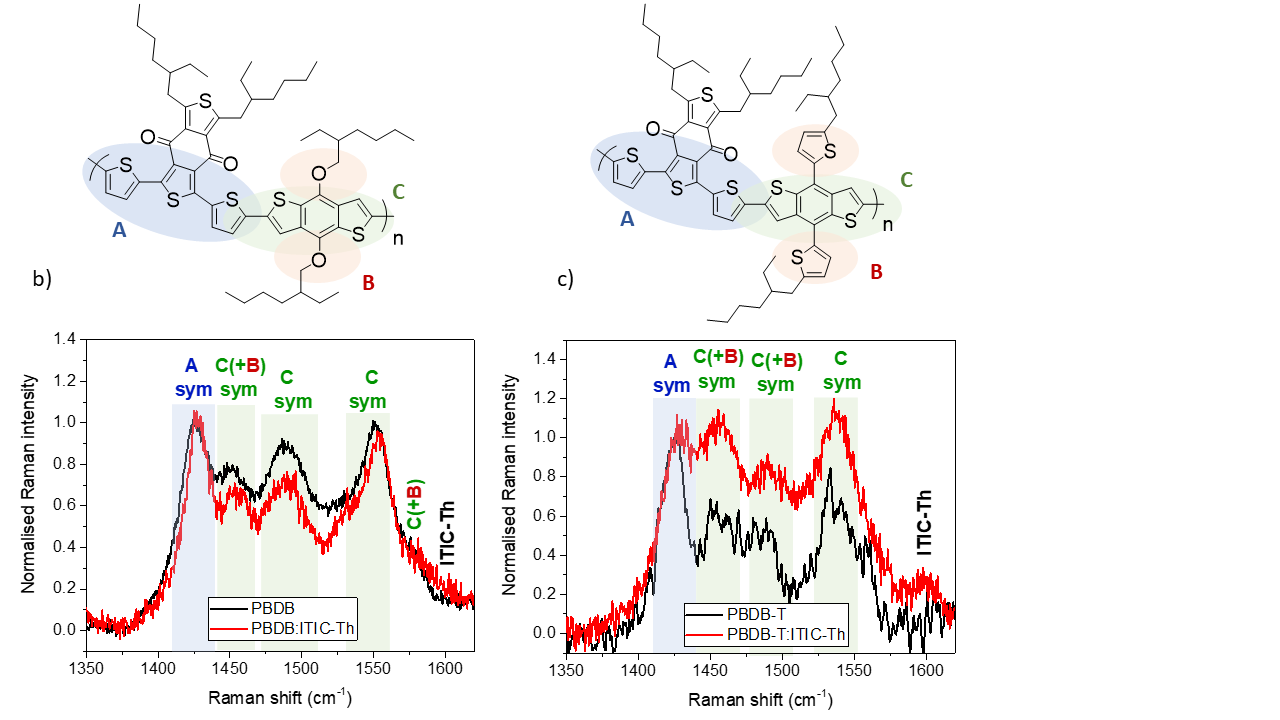

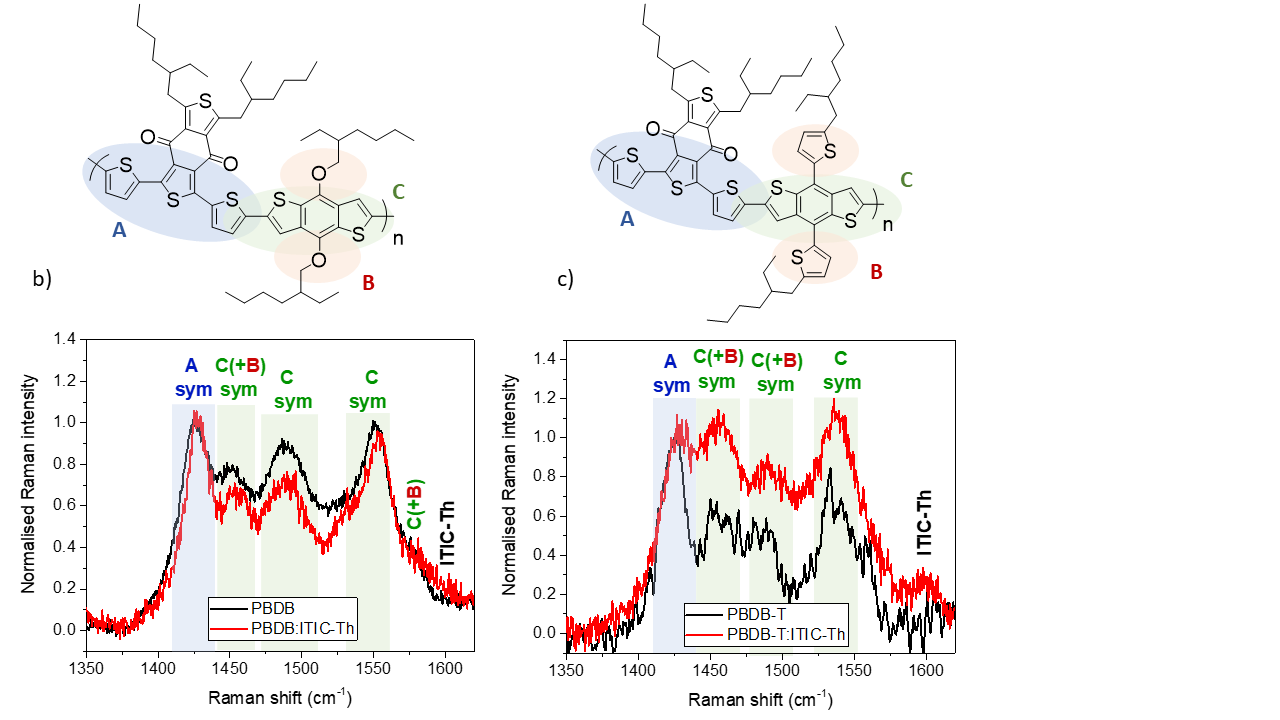


**Table S3.** Raman assignments for PBDB,* with mode numbering matched to Figure S11.

| **Mode** | **Calc (cm^-1^)** | **Exp (cm^-1^)** | **Assignment** |
| --- | --- | --- | --- |
| 1 | 1412 (s) | 1425 (s) | ν_C=C_ (sym) on A thiophenes |
| 2 | 1437 (w) | 1455 (m) | ν_C=C_ (sym) on BDT (C) + ν_C-O_ on B |
| 3 | 1470 (s) | 1486 (m) | ν_C=C_ (sym), BDT (C) |
| 4 | 1508 (m) | 1531 (m) | ν_C=C_ (asym) on A thiophenes + ν_C=C_ (sym) on BDT (C) |
| 5 | 1533 (m) | 1555 (m) | ν_C=C_ (sym) on BDT (C) |
| 6 | 1557 (m) | 1581 (m) | ν_C=C_ (sym) on BDT (C) + δ_C-O_ on B |

* s = strong, m = medium, w = weak; sym = symmetrical, asym = asymmetrical; ν = stretch, δ = bend.

1412 cm^-1^ calc:


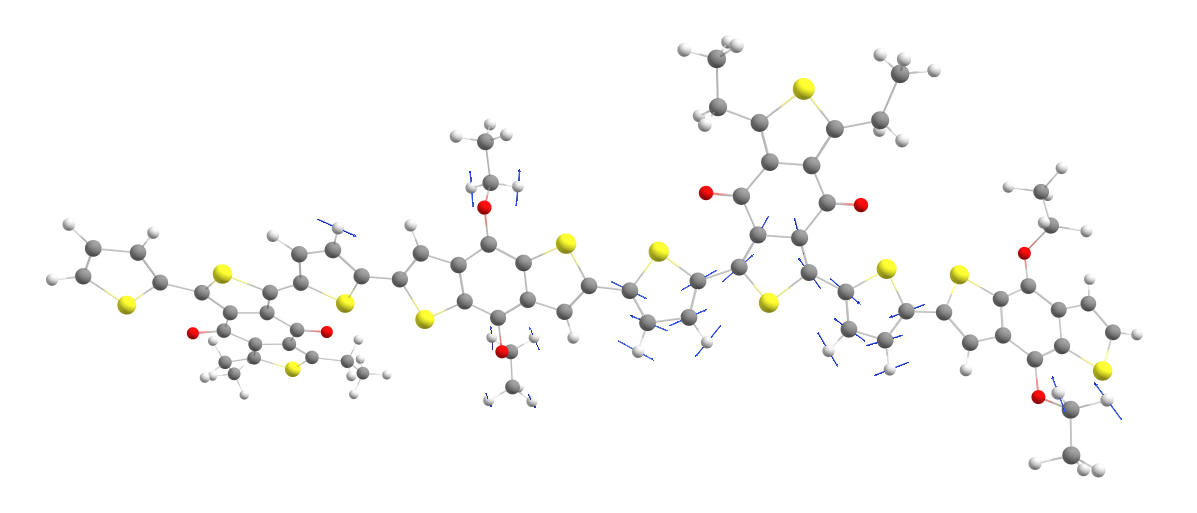


1533 cm^-1^ calc:


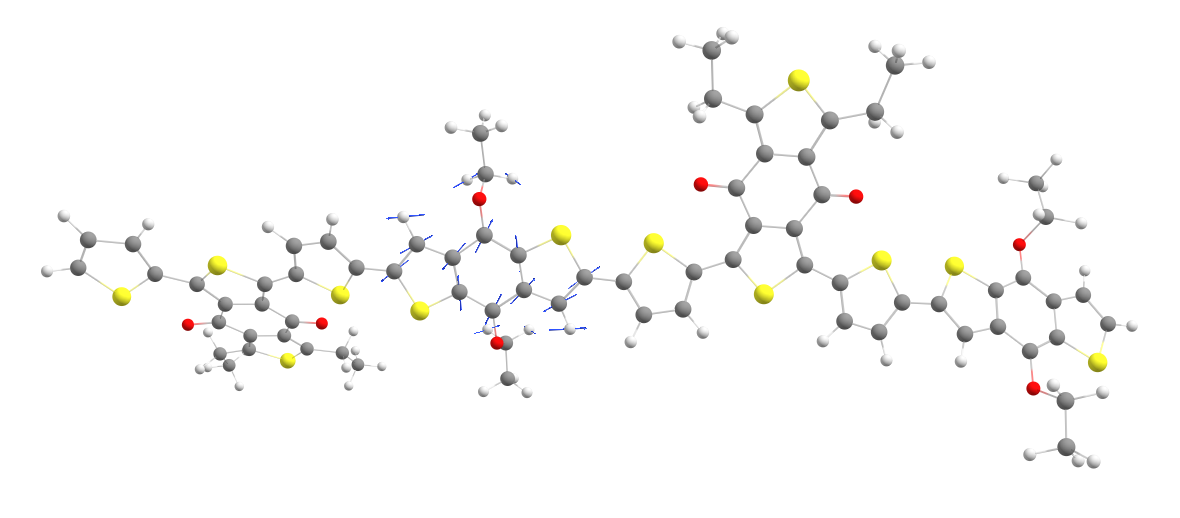


**Figure S12**. The calculated eigenvectors of two PBDB vibrational modes, showing an A-localised mode (1412 cm^-1^) and a C-localised mode (1533 cm^-1^). Calculations were performed using a dimer model with truncated alkyl chains (B3LYP/6-31G(d), frequency scale factor = 0.9613).

**Table S3.** Raman assignments for PBDB-T,* with mode numbering matched to Figure S11.

| **Mode** | **Calc (cm-1)** | **Exp (cm-1)** | **Assignment** |
| --- | --- | --- | --- |
| 1 | 1414 (s) | 1425 (s) | ν_C=C_ (sym) on A thiophenes |
| 2 | 1458 (m) | 1464 (m) | ν_C=C_ (sym) on BDT (C) + ν_C=C_ on B |
| 3 | 1482 (w) | 1489 (m) | ν_C=C_ (sym) on BDT (C) + ν_C=C_ on B |
| 4 | 1505 (m) | 1524 (m) | ν_C=C_ (asym) on BDT (C) + ν_C=C_ (asym) A thiophenes |
| 5 | 1513 (sh) | 1539 (m) | ν_C=C_ (sym) on BDT (C) |
| 6 | 1532 (w) | 1560 (w) | ν_C=C_ (asym) om BDT (C) |

* s = strong, m = medium, w = weak; sym = symmetrical, asym = asymmetrical; ν = stretch, δ = bend.

Calc 1414 cm^-1^:


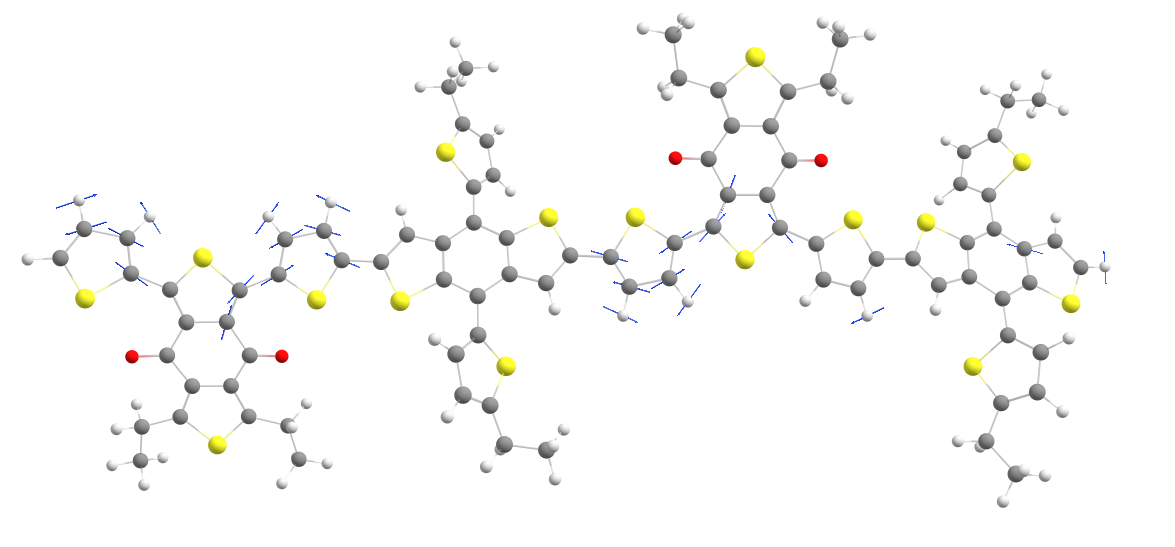


Calc 1458 cm^-1^:


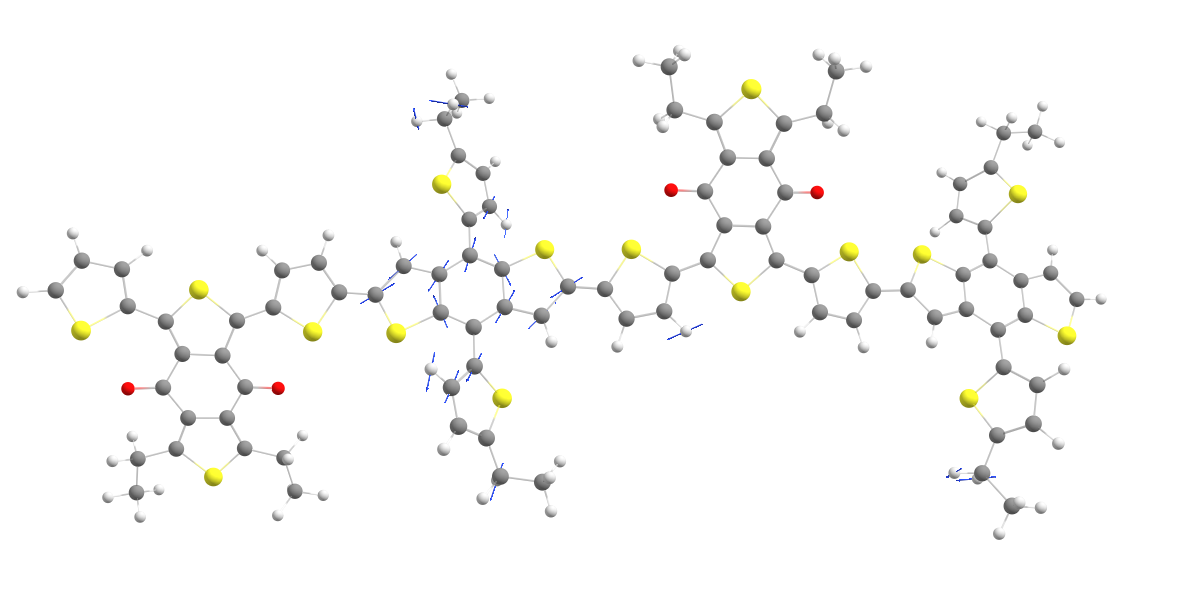


**Figure S13**. The calculated eigenvectors of two PBDB-T vibrational modes, showing an A-localised mode (1414 cm^-1^) and a C-localised mode (1458 cm^-1^). Calculations were performed using a dimer model with truncated alkyl chains (B3LYP/6-31G(d), frequency scale factor = 0.9613).

|  |  |
| --- | --- |
|  |  |

**Figure S14.** Resonance Raman spectra of the pristine polymer films and ITIC-Th blend films as a function of excitation wavelength. In each case, the FT-Raman spectrum of the corresponding pristine polymer powder is shown. Note that the 676 nm resonance Raman spectra of the ITIC-Th blend films shows distinct ITIC-Th peaks (indicated by pink arrows). These get weaker as the excitation wavelength is reduced and the ITIC-Th is no longer in resonance.


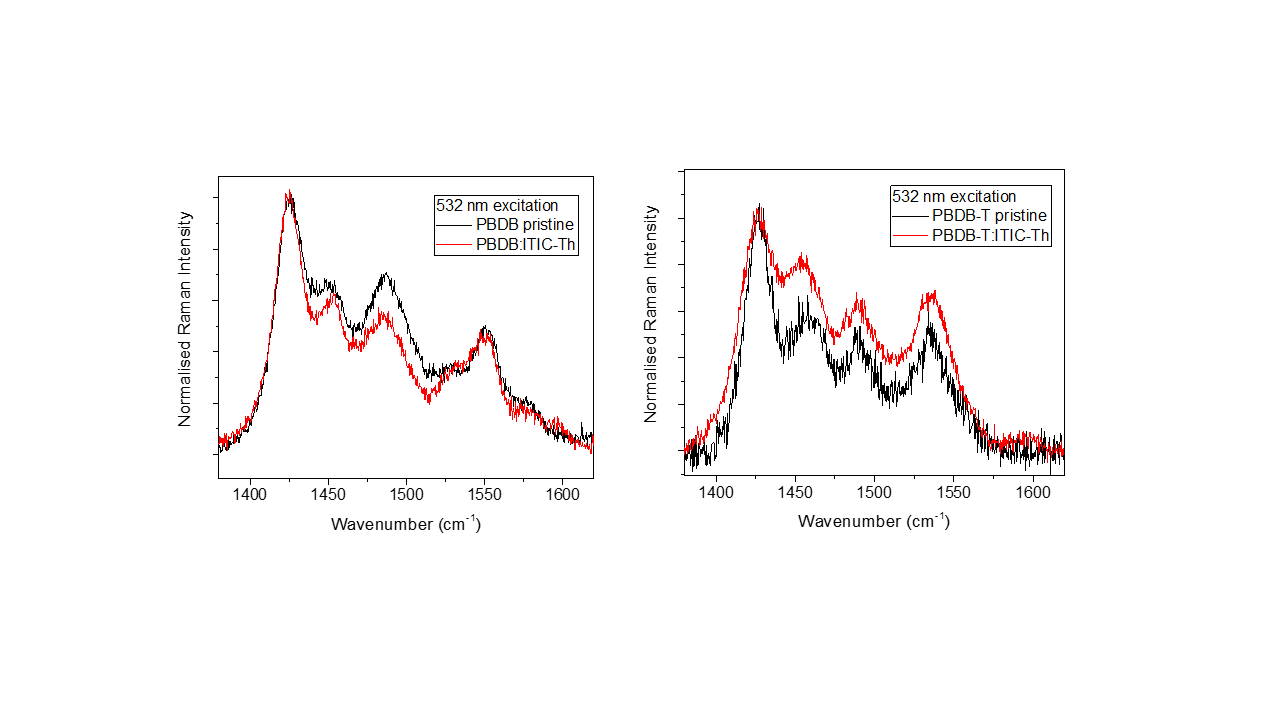


**Figure S15.** The resonance Raman spectra for pristine PBDB and PBDB-T and their blends with ITIC-Th, using an excitation wavelength of 532 nm and a power of 0.2 mW.


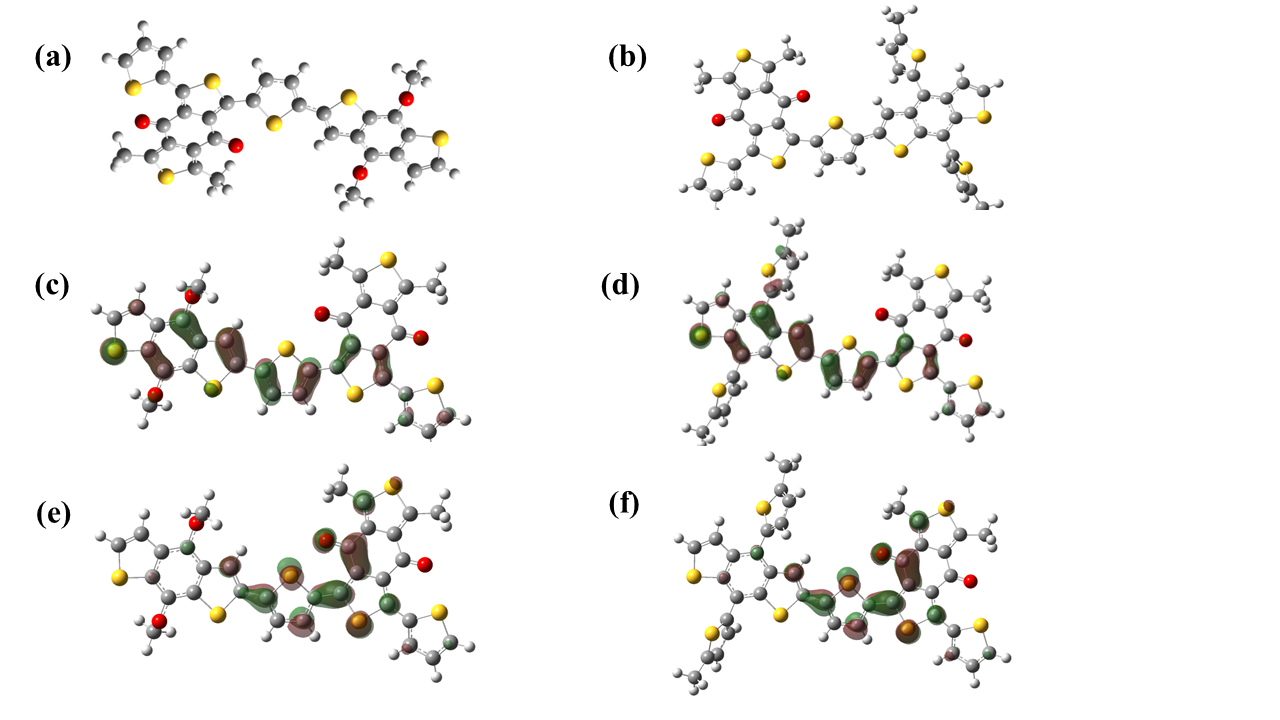


**Figure S16.** The optimised (B3LYP/6-311+G**) geometries of the monomer unit of (a) PBDB and (b) PBDB-T. (c)-(d) The HOMO plots of PBDB and PBDB-T, showing extension of the conjugation onto the peripheral thiophene rings in PBDB-T. (e)-(f) The LUMO plots of PBDB and PBDB-T.


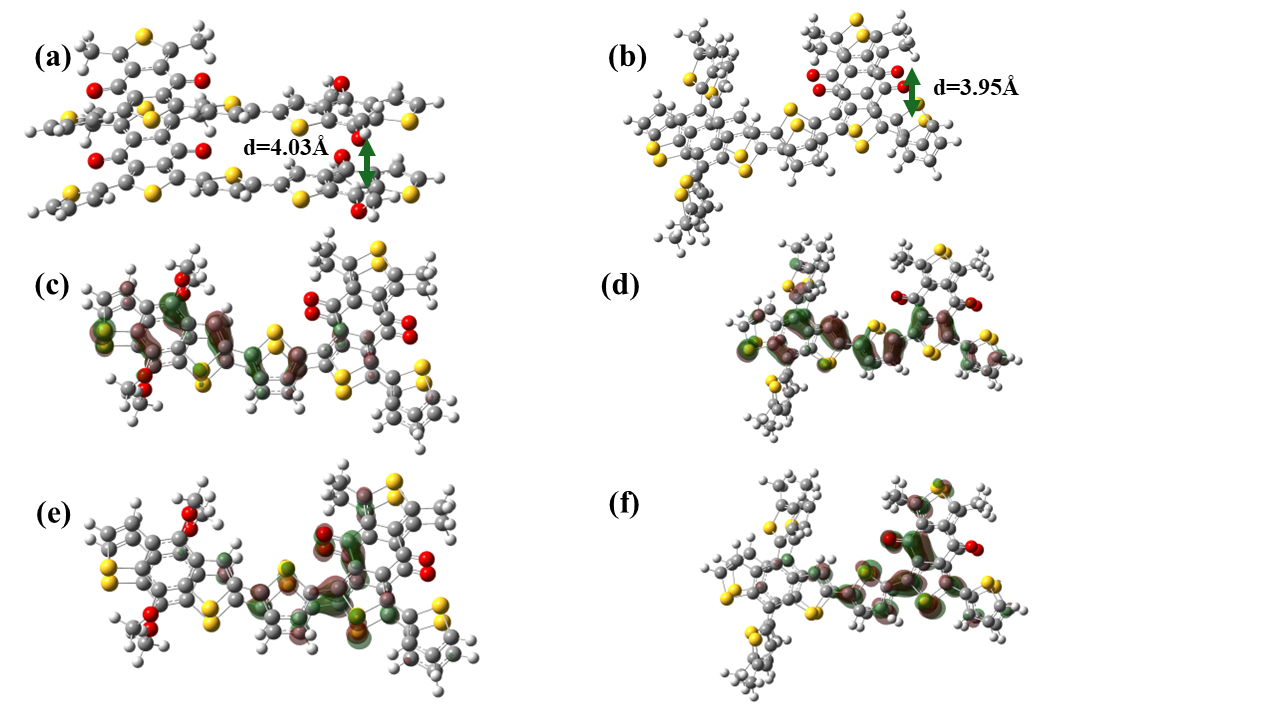


**Figure S17.** (a)-(b) The DFT optimised geometries of the dimer unit of PBDB and PBDB-T. (c)-(d) The HOMO plots of PBDB and PBDB-T. (e)-(f) The LUMO plots of PBDB and PBDB-T.

**References**

1. Ashiotis, G.; Deschildre, A.; Nawaz, Z.; Wright, J. P.; Karkoulis, D.; Picca, F. E.; Kieffer, J., The fast azimuthal integration Python library: pyFAI. *J. Appl. Crystallogr.* **2015,** *48* (Pt 2), 510-519.

2. Marin-Beloqui, J. M.; Congrave, D. G.; Toolan, D. T. W.; Montanaro, S.; Guo, J.; Wright, I. A.; Clarke, T. M.; Bronstein, H.; Dimitrov, S. D., Generating Long-Lived Triplet Excited States in Narrow Bandgap Conjugated Polymers. *JACS* **2023,** *145* (6), 3507-3514.

3. Liang, Y.; Wu, Y.; Feng, D.; Tsai, S.-T.; Son, H.-J.; Li, G.; Yu, L., Development of New Semiconducting Polymers for High Performance Solar Cells. *JACS* **2009,** *131* (1), 56-57.
